# Many lifetime growth trajectories for a single mammal

**DOI:** 10.1002/ece3.8164

**Published:** 2021-10-01

**Authors:** Lara Veylit, Bernt‐Erik Sæther, Jean‐Michel Gaillard, Eric Baubet, Marlène Gamelon

**Affiliations:** ^1^ Department of Biology Centre for Biodiversity Dynamics Norwegian University of Science and Technology Trondheim Norway; ^2^ Université Claude Bernard Lyon 1 Villeurbanne Cedex France; ^3^ Unité Ongulés Sauvages Office Français de la Biodiversité Birieux France

**Keywords:** body growth, development, Gompertz, logistic, monomolecular, *Sus scrofa*

## Abstract

Despite their importance in shaping life history tactics and population dynamics, individual growth trajectories have only been rarely explored in the wild because their analysis requires multiple measurements of individuals throughout their lifetime and some knowledge of age, a key timer of body growth. The availability of long‐term longitudinal studies of two wild boar populations subjected to contrasting environments (rich vs. poor) provided an opportunity to analyze individual growth trajectories. We quantified wild boar growth trajectories at both the population and the individual levels using standard growth models (i.e., Gompertz, logistic, and monomolecular models) that encompass the expected range of growth shapes in determinate growers. Wild boar is a rather altricial species, with a polygynous mating system and is strongly sexually dimorphic in size. According to current theories of life history evolution, we thus expect wild boar to display a sex‐specific Gompertz type growth trajectory and lower sexual size dimorphism in the poorer environment. While wild boar displayed the expected Gompertz type trajectory in the rich site at the population level, we found some evidence for potential differences in growth shapes between populations and individuals. Asymptotic body mass, growth rate and timing of maximum growth rate differed as well, which indicates a high flexibility of growth in wild boar. We also found a cohort effect on asymptotic body mass, which suggests that environmental conditions early in life shape body mass at adulthood in this species. Our findings demonstrate that body growth trajectories in wild boar are highly diverse in relation to differences of environmental context, sex and year of birth. Whether the intermediate ranking of wild boar along the precocial–altricial continuum of development at birth may explain the ability of this species to exhibit this high diversity of growth patterns remains to be investigated.

## Graphical abstract

There is a relative rarity of long‐term studies documenting lifetime body growth trajectories. Using long‐term longitudinal data on two wild boar populations subjected to contrasting environments (rich vs. poor), we demonstrate body growth trajectories in wild boar are context‐, sex‐, and cohort‐specific, differing between populations and among individuals within a population. These findings are novel as they demonstrate that it may be difficult to generalize the shape of a species’ body growth trajectory.

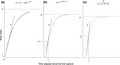

## 1 INTRODUCTION

Body growth trajectories vary widely across species, among populations, and among individuals within a population (Case, 1978; Zullinger et al., 1984). Body growth can be defined as mass change over time (Wellock et al., 2004) as body mass change encompasses both structural size (e.g., skeletal) and condition (e.g., fat reserves). Exploring individual differences in growth trajectories in the wild is challenging because it requires multiple measurements of individuals throughout their lifetime and some knowledge of their age, a key timer of body growth (Reiss, 1989). The availability of long‐term longitudinal studies of ungulate species (Festa‐Bianchet et al., 2017) provides some of the best opportunities to analyze reliably individual growth trajectories, assess among‐individual differences, and identify the role played by the environmental context in shaping the observed growth patterns.

Differences in body growth trajectories among individuals within a population may arise in response to environmental variation and individual differences in energy acquisition and/or allocation. While trade‐offs between body growth and survival or reproduction should exist under our current understanding of life history evolution (Cody, 1966; Stearns, 1992), individual growth is often positively associated with fitness components as the variance in resource acquisition is generally larger than the variance in energy allocation to growth among individuals within a population (van Noordwijk & de Jong, 1986). However, experimental manipulations of reproductive effort have shown that the strength of the trade‐off between growth during development and reproduction varies among individuals (Gélin et al., 2016), which suggests individual differences in allocation strategies. Likewise, resource availability during development (Douhard et al., 2013; McCance, 1962) also influences body growth trajectories. Thus, in environments where resource availability fluctuates, temporal variation in body growth trajectories across individuals should occur. Similarly, there is empirical evidence that harvesting pressure causes variation in growth patterns, with a high hunting pressure favoring faster growth early in life (e.g., Tiilikainen et al., 2010 in moose *Alces alces*). Thus, the environmental context largely determines the growth trajectories of individuals. Differences in body growth trajectories can also be accounted for by sex differences. For instance, in sexually dimorphic and polygynous species, males grow faster and/or for a longer period than females, which leads them to reach a larger asymptotic body mass (e.g., moose *Alces alces* Garel et al., 2006; white‐tailed deer *Odocoileus virginianus* Leberg et al., 1993; Damaraland mole rats *Fukomys damarensis* Zöttl et al., 2016). Finally, across populations, differences in body growth arise due to environmental conditions such as climatic harshness (i.e., thermoregulatory costs in *Alces alces* Sand et al., 1995) and forage availability (*Cervus elaphus atlanticus* Langvatn & Albon, 1986, *Odocoileus virginianus*, Wolverton et al., 2009).

Mammalian growth trajectories have been analyzed using a variety of models depending on the focal species and data available, which range from standard nonlinear growth models (e.g., Gompertz, monomolecular, logistic) to models that are not specific to growth (e.g., state space models, linear regressions). In Table 1, we focus on studies of ungulates because of the high number of published studies based on longitudinal data that capture the full range of growth trajectories in determinate growers.

**TABLE 1:** Studies depicting lifetime growth trajectories in ungulates

Species	Models selected	Sex	Age information	Growth definition	Type of sex‐specific growth trajectory	Reference
*Aepyceros melampus*	Chapman‐Richards, Monomolecular, and Gompertz	M	Y	Body mass vs. age	n/a	Gaillard et al. (1997)
*Aepyceros melampus*	von Bertalanffy	F, M	Y	Body mass and morphological data vs. age	Males grow for longer and reach a larger adult body mass than females.	Howells et al. (1976)
*Alces alces*	Monomolecular	F, M	Y	Carcass mass vs. age	Males grow for longer and reach a larger adult body mass than females.	Solberg et al. (2008)
*Alces alces*	Monomolecular	F, M	Y	Body mass vs. age	Males grow for longer, faster, and reach a larger adult body mass than females.	Garel et al. (2006)
*Alces alces*	Gompertz	F, M	Y	Body mass vs. age	Males grow for longer and reach a larger adult body mass than females.	Sand et al. (1995)
*Bison bison*	Logistic	F	Y	Body mass and morphological data vs. Age	n/a	Green and Rothstein (1991)
*Bos javanicus*	von Bertalanffy	n/a	Y	Body mass vs. age	n/a	Hafiz et al. (2016)
*Capra aegagrus hircus*	Weibull	F	Y	Body mass and morphological data vs. age	n/a	Kor et al. (2011)
*Cervus elaphus*	von Bertalanffy	F	Y	Body mass and morphological data vs. age	n/a	Bertouille and de Crombrugghe (1995)
*Cervus elaphus*	n/a	F, M	Y	Body mass vs. age	Males grow for longer and reach a larger adult body mass than females.	Jarman (1983), Mitchell et al. (1977)
*Cervus nippon yesoensis*	von Bertalanffy	F, M	Y	Body mass and morphological data vs. age	Males grow for longer and reach larger adult body mass than females.	Suzuki et al. (2001)
*Kobus ellipsiprymnus*	n/a	F, M	Y	Body mass vs. age	Males grow for longer and reach larger adult body mass than females.	Jarman (1983), Spinage (1969)
*Kobus leche*	n/a	F, M	Y	Body mass vs. age	Males grow for longer and reach larger adult body mass than females.	Jarman (1983), Robinette and Child (1964)
*Madoqua kirkii*	n/a	n/a	n/a	n/a	Both sexes grow at a similar rate before maturity and reach a similar adult body mass.	Jarman (1983), Kellas (1955)
*Muntiacus reevesi*	Logistic	F, M	Y	Carcass mass and morphological data vs. age	Season of birth did not influence time to reach sexual maturity in females, season of birth influenced time to reach sexual maturity in males	Chapman et al. (1997)
*Odocoileus virginianus*	von Bertalanffy	M	Y	Body mass and morphological data vs. age	n/a	Monteith et al. (2009)
*Odocoileus virginianus*	Richards	F, M	Y	Body mass vs. age	n/a	Leberg et al. (1989)
*Odocoileus virginianus*	Richards	F, M	Y	Body mass vs. age	Males grew slower and reached smaller adult body masses in high population densities. Females do not have a strong growth‐density relationship.	Leberg and Smith (1993)
*Ovibos moschatus*	Gompertz	n/a	Y	Body mass and morphological data vs. age	n/a	Knott et al. (2005)
*Ovis aries*	State space model	F	Y	Body mass vs. age	n/a	Brooks et al. (2017)
*Ovis aries*	Linear regression	F	N	Body mass at time t vs. body mass at time t + 1	n/a	Coulson (2012)
*Ovis canadensis*	Linear regression	F, M	N	Body mass at time t vs. body mass at time t + 1	Males grow for longer and reach larger adult body mass than females.	Traill et al. (2014)
*Ovis canadensis*	Lopez	F	Y	Body mass vs. age	n/a	Marcil‐Ferland et al. (2013)
*Ovis canadensis*	Linear regression	F, M	N	Body mass vs. days from a set date	Males had higher seasonal mass gain than females. Males grew for a longer period than females.	Festa‐Bianchet et al. (1996)
*Rangifer tarandus*	Gompertz	n/a	Y	Body mass and morphological data vs. age	n/a	Knott et al. (2005)
*Sus scrofa domesticus*	Lopez	F, M	Y	Body mass vs. age	Males grow for longer than females. Males did not reach their adult body mass.	Strathe et al. (2010)
*Sus scrofa domesticus*	Logistic function	F, M	Y	Body mass vs. age	Both sexes grow at a similar rate initially. Males then grow at a higher rate after early life.	Vincek et al. (2012)
*Sus scrofa domesticus*	n/a	F, M	Y	Body mass vs. age; Morphological data vs. age	Both sexes grow at a similar rate initially. Males then grow at a higher rate than females.	Walstra (1980)
*Sus scrofa domesticus*	Gompertz	M	Y	Body mass vs. age	n/a	Ceron et al. (2020)
*Sus scrofa*	Linear regression	F, M	Y	Body mass and morphological data vs. age	Males grow for longer than females. Males did not reach their adult body mass.	Gallo Orsi et al. (1995)
*Sus scrofa*	n/a	F, M	Y	Body mass vs. age	Both sexes grew at around the same rate until 1 year of age, then males grew faster than females.	Pépin (1991)
*Sus scrofa*	Polynomial growth curve	F, M	Y	Body mass vs. age	Males grew slower and for a longer period than females.	Pedone et al. (1995)
*Sylvicapra grimmia*	Gompertz	F, M	Y	Body mass vs. age	n/a	Gaillard et al. (1997)
*Syncerus caffer*	n/a	F, M	Y	Body mass vs. age	Males and females grow throughout life, females have a lower growth rate than males.	Jarman (1983), Sinclair (1977)
*Taurotragus oryx*	Gompertz	F, M	Y	Body mass vs. age	n/a	Gaillard et al. (1997)

Note

The “model selected” specifies which model(s) performed best for each “species.” Information is provided on whether data came from males (M) and/or females (F). Whether the model required age‐specific data is given in “age information.” The type of data used to model growth in the study is given in “growth definition.” When information on growth trajectories of both sexes was available, the “type of sex‐specific growth trajectory” is reported. The literature survey was conducted by searching the terms “body growth AND ungulate,” “body growth trajectory AND ungulate,” ISI Web of Science, Google Scholar, and by searching the references in relevant papers. Only papers that documented growth through adulthood were retained. This is a noncomprehensive list of studies due to the breath of literature on body growth across fields. The search was conducted in September 2020.

**FIGURE 1:**
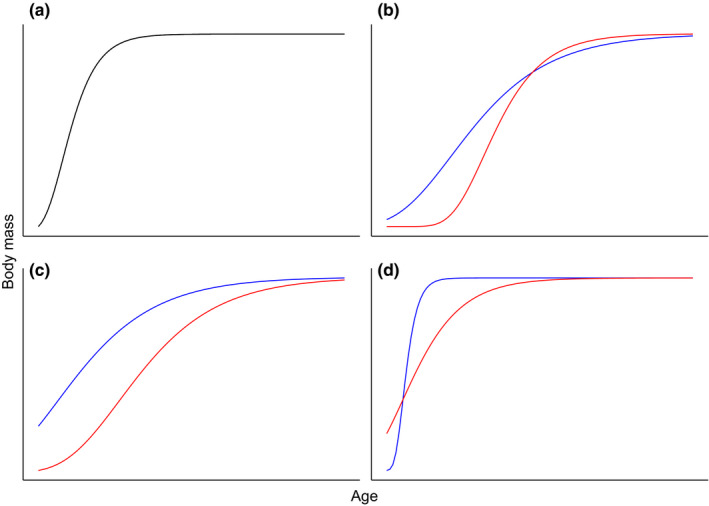
Four scenarios of sex‐specific growth trajectories (red = male, blue = female). (a) Both sexes exhibit the same growth rate and the same duration of the growth period, which leads both sexes to show the same growth trajectory. (b) Males and females have different growth rates and different duration of the growth period; (c) Males and females exhibit the same growth rate but have different duration of the growth period; (d) Males and females have different growth rates but the same duration of the growth period. For the sake of simplicity, we constrained the asymptotic body mass for males and females to be the same in all scenarios

While the growth process is defined as changes in mass over time, most empirical studies on mammalian growth have focused on the age‐specific changes in mass (e.g., Gaillard et al., 1997; Howells, 1976). In studies based on statistical models not specific to body growth (e.g., linear regressions, state space models), growth can be measured by different parameters (e.g., seasonal mass gain Festa‐Bianchet et al., 1996; residuals of relationship between body mass and age at capture Plard et al., 2015) without specification of the growth shape. Most often, in comparative analyses of growth, different growth models are fitted and compared (e.g., Gaillard et al., 1997; Leberg et al., 1989) across species when ecological or life history correlates are looked for. For example, precocial species typically exhibit a monomolecular growth shape involving a consistently decreasing growth rate from birth onwards (Gaillard et al., 1997; but see English et al., 2012 for an altricial species with this type of growth trajectory). On the other hand, altricial species generally display a sigmoidal growth shape (Gompertz) involving a maximum growth rate that occurs during the postnatal period (Gaillard et al., 1997). Likewise, the magnitude of sex differences in growth trajectories varies considerably across species (see Table 1) and can follow four major types. First, in monomorphic species, females and males grow at similar rates for the same time period (Figure 1a). Second, males and females differ in both growth rates and duration of the growth period. For instance, females grow rapidly in early life and reach quickly their asymptotic body mass, leading them to grow faster but for a shorter period than males (Figure 1b). Third, both sexes can exhibit similar growth rates but one sex has a shorter growth period (Figure 1c). Lastly, one sex grows faster (usually males in ungulates) but both sexes have the same duration of the growth period (Figure 1d).

Taking advantage of long‐term individual monitoring data, we compared sex‐specific growth trajectories for two wild boar (*Sus scrofa*) populations subject to markedly different environmental conditions. One population (Chizé) was subject to a weak hunting pressure and faced with food‐limited conditions, while the other (Châteauvillain) was subject to a strong harvesting pressure and had access to abundant food resources. Previous studies of wild boar growth trajectories focused on single populations and were based on statistical models not specific to growth (e.g., polynomial curve in Pedone et al., 1995; linear regressions in Gallo Orsi et al., 1995; linear approximation of early‐life growth in Gaillard et al., 1993). Growth of domestic pigs (*Sus scrofa domesticus*) has been investigated using growth models fitted to age‐specific data of body mass change. These models showed that pig growth follows a sigmoidal function (e.g., Strathe et al., 2010; Vincek et al., 2012) and that the Gompertz model might be the most suitable (Ceron et al., 2020). Here, first, we quantified growth trajectories for each sex in each population (i.e., testing for context‐specific growth trajectories) using Gompertz, logistic, and monomolecular models, which encompass the expected range of growth shape in determinate growers. We tested for differences in adult body mass, maximum growth rate, and time required to reach maximum growth across individuals. Because wild boars are rather altricial (i.e., are born in a nest and are highly restricted in their movements in their first days of age) and not as precocial as similar‐sized ungulates (Gaillard et al., 1997), and in accordance with recent growth modeling in pigs (Ceron et al., 2020), we thus expected wild boar to exhibit a Gompertz type growth trajectory. Moreover, as wild boars are strongly sexually dimorphic in size (Toïgo et al., 2008) with similar growth early in life for both sexes (Gaillard et al., 1992; Veylit et al., 2020b), we expected sex‐specific growth trajectories to follow our third scenario (i.e., males and females have the same growth rate but different growth period durations; Figure 1c). Therefore, we expected males and females to grow at the same rate but for males to grow for a longer period to reach a larger asymptotic body mass than females. As the amount of sexual size dimorphism decreases with increasingly harsh environmental conditions in polygynous mammals (e.g., Leblanc et al., 2001), we expected stronger sex differences to occur in the heavily harvested site with regular access to high‐quality resources. Second, we tested for differences in asymptotic body mass among cohorts in both sexes. We expected individuals born in different years to differ in adult body mass. Lastly, we used a dataset restricted to individuals with multiple body mass measurements early and late in life for which the full growth trajectories could be assessed. On this restricted dataset, we determined the best model for each individual's body growth trajectory by fitting body growth models to individual data. By doing so, we tested whether individuals of a given sex and site exhibit the same type of growth trajectory (expected to be the Gompertz type) or whether individual heterogeneity in growth trajectory exists. As wild boars exhibit high variation in body growth rates during early life (Veylit et al., 2020b), we expected a high variation in body growth trajectories across individuals to occur in both study areas.

## 2 MATERIALS AND METHODS

### 2.1 Study sites and data collection

The study was conducted in two French wild boar populations subject to contrasting environments. The population in the 11,000 ha forest of Châteauvillain in northeastern France (48.02°N, 4.56°E) is heavily harvested (on average 727.18 ± 282.07 individuals shot per year, see Veylit et al., 2020b), with hunting being oriented toward young individuals (juveniles, see Gamelon et al., 2011). The forest is characterized by a climate intermediate between continental and oceanic and dominated by beech (*Fagus sylvatica*) and oak (*Quercus* spp.), which produce preferred forage for wild boar (Gamelon et al., 2017; Servanty et al., 2011; Touzot et al., 2020). The second population is found in the 2,614 ha Réserve Biologique Intégrale at Chizé in southwestern France (46.05°N, 0.25°W), characterized by mild winters and often warm, dry summers. As the soil in Chizé is of poor quality and the site is subject to frequent summer droughts, the forest productivity is low (Pettorelli et al., 2006). The site is therefore considered of poor quality (Douhard et al., 2013; Gaillard et al., 2003). The population in Chizé is subject to a light hunting pressure (on average 101.50 ± 80.94 individuals shot per year, see Veylit et al., 2020b).

In both sites, a capture–mark–recapture–recovery (CMRR) program allows for capturing, marking using traps, then releasing wild boars each year between March and September since >30 years. Additionally, between October and February, individuals are removed each year from both populations by either hunting or translocation. Sex, date, and body mass to the nearest 0.1 kg are recorded for each individual first caught below 20 kg (i.e., younger than 6 months of age) and later on, during subsequent captures (alive and dead from hunting). Based on tooth eruption patterns, the youngest animals trapped were 3 months of age (Gamelon et al., 2011). Only measurements collected more than seven days apart were included in the analyses (see Veylit et al., 2020a).

### 2.2 Growth trajectories at the population level

We explored site‐ and sex‐specific growth patterns. Importantly, the exact age was not available, which prevented us from assessing body growth trajectories using the commonly used mass–age relationship. Instead, we modeled body mass as a function of time elapsed from the first capture. All individuals were first captured within their 6 first months of life (i.e., below 20 kg, Gaillard et al., 1992; Veylit et al., 2020a). We only retained individuals with at least three body mass measurements, including two measurements taken in the first 6 months of age (i.e., below 20 kg) to assess the early‐life growth rate during the stage when growth is linear (Gaillard et al., 1992; Veylit et al., 2020b), and one measurement taken later in life (i.e., above 20 kg) to assess body growth later in life. In Châteauvillain and Chizé, there was an average of 411.46 (range 36–2722) and 608.70 (range 42–2052) days, respectively, between the first and the last captures (see Supporting Information S1 for time from first capture for each mass measurement).

**FIGURE 2:**
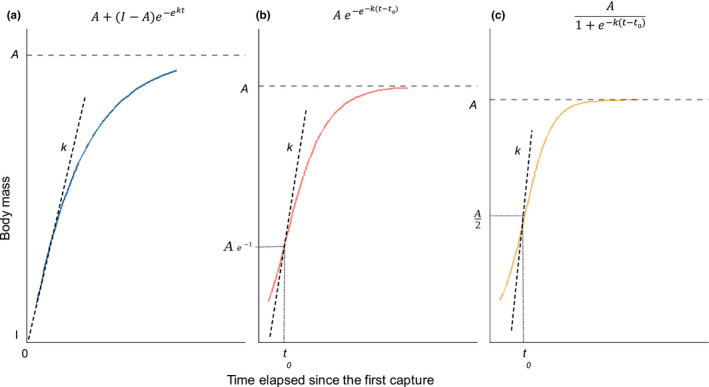
Growth functions used to model wild boar body growth trajectories: (a) monomolecular; (b) Gompertz; (c) logistic. *A* is the asymptotic body mass (kg) and *k* is the relative growth rate (days^−1^). For monomolecular model, *I* corresponds to the body mass at first capture. For Gompertz and logistic models, *t*_0_ is the time (in days from first capture date) of maximum growth and is located at the inflection point

The three equations used to model body growth (monomolecular, Gompertz, and logistic models) are adapted from Gaillard et al. (1997), Zullinger et al. (1984), and English et al. (2012) (see Figure 2). These equations are characterized by three main parameters: the asymptotic body mass (*A*, in kg), the relative growth rate (*k*, in days^−1^), and *t*_0_ (in days) for Gompertz and logistic models. For the biological interpretation of the relative growth rate *k*, in the case of a Gompertz function, *k* can be converted to maximum growth rate *K* (in kg days^−1^) by multiplying *k* by *A × e^−^* ^1^ (estimated body mass at the inflection point, see Figure 2b). Likewise, for a logistic function, *k* can be converted to maximum growth rate *K* (in kg days^−1^) by multiplying *k* by *A/2* (estimated body mass at the inflection point, see Figure 2c). For the monomolecular function, *k* can be converted to maximum growth rate *K* (in kg days^−1^) by multiplying *e^k^* by *A*. The parameter *t_0_* is interpreted as the time required to reach maximum growth. It occurs at about 37% and 50% of the mature body mass (asymptote) for Gompertz and logistic functions, respectively. For monomolecular function, *I* corresponds to the mean body mass at first capture.

We fitted nonlinear mixed effects models to account for multiple measures for a given individual by including individual identity as a random effect. However, due to convergence issues, it was not possible to account for repeated mass measurements on all parameters (i.e., *A*, *k* and *t*_0_) simultaneously. We thus tested models with individual identity as a random effect either on *A*, *t_0_* or *k* values. In addition, we explored the variation in *A*, the asymptotic body mass, in relation to year of first capture (i.e., birth year) to assess the magnitude of cohort effects. Indeed, conditions experienced in early life may influence adult body mass of all individuals born within the same year in a similar way. To do so, we included year of first capture (birth year) as a categorical factor influencing *A* values (see Supporting Information S2 for distributions of years of first capture). We used the nlme package (v. 3.1‐140, Pinheiro et al., 2020) in R v. 3.6.0 (R Development Core Team, 2020) and AIC to identify the best model (Burnham & Anderson, 2002). Models within two AIC were considered to perform similarly, and following the rules of parsimony, the model with fewer parameters was selected. Analyses were conducted on 465 individuals (248 males, 217 females) captured between 1983 and 2016 at Châteauvillain and on 156 individuals (83 males, 73 females) captured between 2003 and 2016 at Chizé (see Supporting Information S3 for distributions of number of mass measurements for each site).

### 2.3 Individually fitted body growth models

Taking advantage of the high‐quality long‐term data we had available, we then reduced our dataset to only the individuals that were weighed the last time at least 2 years after the first measurement and had more than two mass measurements at or above 20 kg (i.e., older than 6 months). This allowed us to explore growth trajectories for the oldest individuals in the datasets that have likely reached their asymptotic body mass. Contrary to population‐level analyses, this analysis not only allowed us to estimate individually varying parameter values (e.g., *k*, *A*), but also allowed us to test for different body growth shapes (Gompertz, logistic, or monomolecular) for each individual. We thus identified which model (i.e., Gompertz, logistic, monomolecular; see Figure 2) best fit each individual's body growth trajectory. We conducted model selection for each individual using AIC. From the best model retained, we recorded *A*, *t*_0_ (for Gompertz and logistic models), *I* (for monomolecular models), and *k* values for each individual. We then determined the proportion of individuals each model fit best for each sex and site (R codes used for all analyses are provided in Supporting Information S4). We also compared parameters (i.e., *A*, *k*, *t*_0,_ and *I*) among sites and sexes using *t*‐tests (Supporting Information S9). In Châteauvillain, this restricted dataset resulted in 37 individuals (29 females, 8 males) with an average of 6 measurements per individual (range 4–13). In Chizé, we used data from 12 individuals (4 females, 8 males) with an average of 4 measurements per individual (range 4–5).

## 3 RESULTS

### 3.1 Growth trajectories at the population level

**FIGURE 3:**
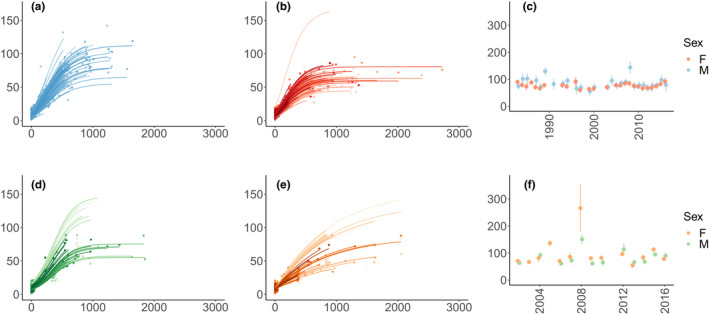
Body growth trajectories at Châteauvillain (first row) and Chizé (second row), for males (panels a, d) and females (panels b, e). Points depict observations with matching colors of individual growth curves, from the selected models (see models retained in Table 2). Panels c and f show asymptotic body mass *A* according to birth year for both sexes (i.e., cohort effect on *A*)

In Châteauvillain, the average body growth of wild boar displayed a Gompertz type trajectory (Table 2), for both sexes. This model included a random intercept of individual identity on adult body mass *A*. It also included birth year as a categorical effect on *A,* indicating that the asymptotic body mass depended on the year of birth in both sexes (Figure 3c). Noticeably, this cohort effect on asymptotic body mass was similar for males and females; that is, we observed similar fluctuations of asymptotic body mass in both sexes across cohorts (Figure 3c). The asymptotic body mass fluctuated between 70.41 kg (for the cohort born in 1999) and 158.89 kg (for the cohort born in 2008) for males and between 48.54 kg (for the cohort born in 1997) and 80.56 kg (for the cohort born in 1996) for females. For males, the relative growth rate *k* was 1.42 year^−1^ (*SE*: 0.03) indicating a maximum growth rate *K* of 100.77 g day^−1^ for the cohort 1997 and a maximum growth rate *K* of 227.40 g day^−1^ for the cohort 2008. For females, *k* was 1.81 year^−1^ (*SE*: 0.04) corresponding to a maximum growth rate *K* of 88.55 g days^−1^ for the cohort 1997 and 146.96 g days^−1^ for the cohort 1996. Maximum growth rates occurred 233.6 days (*SE*: 6.57) after the first capture for males and 149.7 days (*SE*: 4.02) after the first capture for females (Figure 3a,b). Thus, in Châteauvillain, males grew for a longer period, reached a heavier asymptotic body mass, and grew with higher maximum rates than females.

**TABLE 2:** Comparison of the average individual growth trajectory of wild boar from populations in Châteauvillain and Chizé, France, for males (M) and females (F)

Model	Random effect	Categorical effect	Châteauvillain		Chizé	
			AIC (M)	AIC (F)	AIC (M)	AIC (F)
Gompertz	None	None	21,861.92	19,143.34	6,194.59	5,260.54
Gompertz	*A*	None	21,170.05	18,744.58	6,081.43	5,151.41
Gompertz	*t*_0_	None	21,767.98	19,122.09	6,184.61	5,261.71
Gompertz	*k*	None	21,849.98	19,146.57	6,193.33	5,262.54
Gompertz	*A*	Birth year	***21,132.26***	***18,726.56***	6,031.42	5,105.24
Gompertz	*t*_0_	Birth year	21,447.60	18,967.60	6,113.43	5,125.22
Gompertz	*k*	Birth year	21,585.13	19,003.24	6,110.85	5,142.66
Logistic	None	None	21,913.96	19,208.65	6,178.70	5,270.13
Logistic	*A*	None	21,289.52	18,808.17	6,080.90	5,159.02
Logistic	*t*_0_	None	21,783.29	19,185.36	6,153.19	5,270.76
Logistic	*k*	None	21,911.02	19,210.65	6,180.73	5,272.35
Logistic	*A*	Birth year	21,254.26	18,788.78	***6,028.93***	5,118.61
Logistic	*t*_0_	Birth year	21,542.91	19,036.42	6,100.26	5,139.29
Logistic	*k*	Birth year	21,649.94	19,074.37	6,100.18	5,151.07
Monomolecular	None	None	21,921.51	19,124.34	6,222.41	5,254.10
Monomolecular	*A*	None	NA	18,806.67	NA	NA
Monomolecular	*k*	None	21,899.45	19,115.58	6,224.41	5,256.10
Monomolecular	*A*	Birth year	NA	18,790.45	6,051.51	***5,095.44***
Monomolecular	*k*	Birth year	21,600.54	18,951.09	6,136.50	5,104.86

Note

Models include individual random intercepts on asymptotic body mass *A*, timing of the maximum growth *t*_0_ (for Gompertz and logistic models) or relative growth rate *k*. Birth year is included as a categorical effect to test for potential cohort effects on *A*. The best model for each sex and site with the lowest AIC is indicated in bold. NA means that the model did not converge.

At Chizé, males displayed an average body mass growth trajectory best described by a logistic model with an individual random intercept on asymptotic mass (Table 2, Figure 3d). As found in Châteauvillain, the best model included birth year as a categorical effect on *A* indicating that the asymptotic body mass for males also depended on the year of birth (Figure 3f). It fluctuated between 61.40 kg (for the cohort born in 2006) and 151.61 kg (for the cohort born in 2008). The relative growth rate *k* was estimated to 2.10 year^−1^ (*SE*: 0.09) corresponding to a maximum growth rate *K* of 176.63 g.day^−1^ for the cohort 2006. The maximum growth rate occurred 350.4 days (*SE*: 19.71) after the first capture, thus later than in Châteauvillain. Females displayed an average body mass growth trajectory best described by a monomolecular model with an individual random intercept on asymptotic mass (Table 2, Figure 3e). As for males, the asymptotic body mass for females depended on the year of birth (Figure 3f). It fluctuated between 50.88 kg (for the cohort born in 2013) and 262.23 kg (for the cohort born in 2008). Note that this last value is biologically unrealistic and is associated with large uncertainty (see Figure 3f). For the monomolecular model, *k* was estimated as −0.91 (*SE*: 0.12) thus corresponding to a maximum growth rate at first capture *K* of 56.11 g day^−1^ for the cohort born in 2013. The mean body mass at first capture, *I*, was estimated as 7.73 kg (*SE*: 0.35). Therefore, as observed in Châteauvillain, males grew with higher maximum rate and reached a larger asymptotic body mass than females. Maximum growth rates and asymptotic body mass were lower in Chizé than in Châteauvillain.

### 3.2 Individual growth trajectories

We fit each model (monomolecular, Gompertz, and logistic) to each individual's data, allowing the assessment of individual‐specific growth trajectory for those individuals with multiple measurements later in life.

**FIGURE 4:**
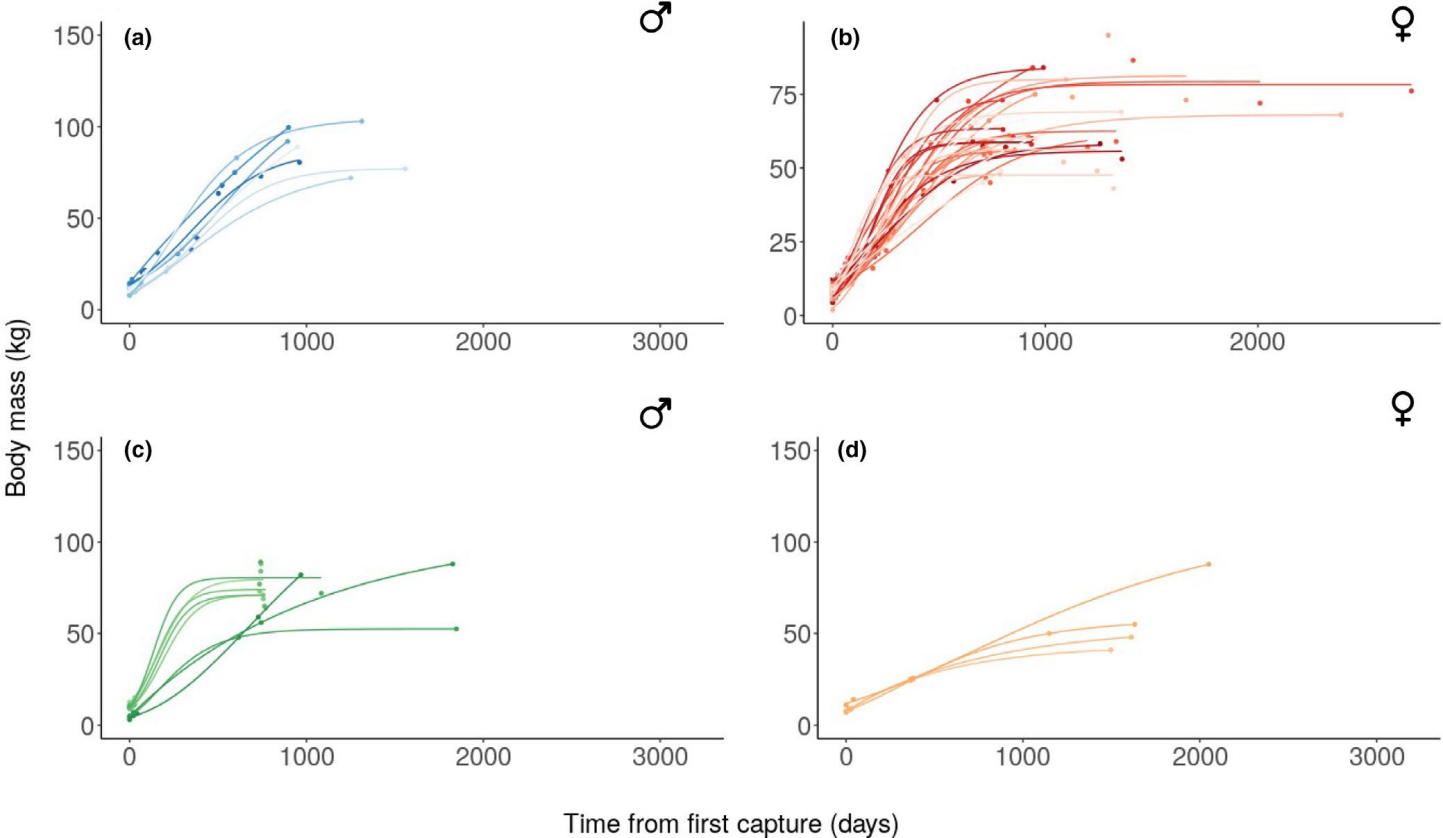
Body growth trajectories at Châteauvillain (first row) and Chizé (second row), for males (panels a, c) and females (panels b, d), from the dataset restricted to individuals with repeated measurements during both early and late in life. Points depict observations with matching colors to the corresponding individual's body growth curve that best fit the data (either monomolecular, Gompertz, or logistic, see Supporting Information S5–S8)

We found that males in Châteauvillain displayed a body mass growth trajectory best described by a Gompertz (50%) or a logistic (38%) model and at a lesser extent, by monomolecular models (13%), demonstrating a high diversity of body growth trajectories among males within this population (Table 3, Figures 4a and S5). We also found a high variation in asymptotic body mass *A*, timing of the maximum growth *t*_0_ (for Gompertz and logistic models) and relative growth rate *k* across individuals. For females, the logistic model performed best for most individuals (66%), followed by the Gompertz (24%) and the monomolecular models (10%) (Table 3, Figure 4b, Supporting Information S6). As observed for males, we also found a high variation in asymptotic body mass *A*, timing of the maximum growth *t_0_* (for Gompertz and logistic models) and relative growth rate *k* across females. In accordance with the results obtained at the population level (see above, Section 3.1), males consistently grew for a longer period and reached a larger asymptotic body mass than females.

**TABLE 3:** Sex‐ and site‐specific proportion of individuals following a body growth trajectory best fitted by Gompertz, logistic or monomolecular models (see Section 2.3 for sample sizes)

	Châteauvillain		Chizé	
	Males	Females	Males	Females
Gompertz				
Proportion	0.50	0.24	0.25	0.50
*A*	108.03 [78.63, 126.94]	67.43 [48.50, 84.06]	99.00 [52.53, 145.46]	84.03 [57.56, 110.51]
*k*	1.09 [0.72, 1.45]	2.00 [1.23, 2.55]	1.31 [0.69, 1.93]	0.63 [0.40, 0.85]
*t*_0_	311.12 [233.33, 427.23]	166.04 [101.55, 241.79]	419.04 [165.02, 673.05]	514.14 [302.93, 725.34]
Logistic				
Proportion	0.38	0.66	0.63	0.00
*A*	93.36 [77.25, 116.49]	66.01 [47.60, 81.22]	75.30 [71.07, 80.50]	
*k*	1.64 [1.37, 1.80]	2.89 [1.50, 5.64]	4.33 [3.76, 5.31]	
*t*_0_	428.77 [351.08, 541.16]	271.88 [92.47, 428.63]	161.45 [135.66, 189.11]	
Monomolecular				
Proportion	0.13	0.10	0.13	0.50
*A*	250.23	99.29 [84.23, 108.96]	105.68	48.49 [43.49, 53.50]
*k*	−1.71	−0.94 [−1.40, −0.60]	−1.06	−0.58 [−0.73, −0.43]
*I*	14.73	8.59 [7.08, 9.81]	4.74	7.40 [7.16, 7.63]

Note

Displayed are the average estimated asymptotic body mass *A* (in kg), timing of the maximum growth *t*_0_ (in days) for Gompertz and logistic models, mean body mass at first capture *I* (in kg) for monomolecular models, and relative growth rate *k* (in years^−1^) (mean [min; max] or only mean when there is a single individual).

At Chizé, the eight males included in the analysis displayed a body mass growth trajectory best described by a logistic model (63%) and at a lesser extent by Gompertz (25%) and monomolecular models (13%) (Table 3, Figure 4c, Supporting Information S7), whereas Gompertz and monomolecular models provided equal best fit (50%) for the four females of the analysis (Table 3, Figure 4d, Supporting Information S8). These findings again highlight a high diversity of body growth trajectories for a given sex in a given site.

In addition to finding a diversity of body mass growth trajectories among individuals, we found, for a given growth shape (e.g., Gompertz), a diversity of parameter values (i.e., *A*, *k, t*_0_ and *I*) across sexes and sites. In Châteauvillain, among individuals that displayed a body growth trajectory best described by a Gompertz model, we detected sex differences in asymptotic body mass *A*, relative growth rate *k* and timing of maximum growth *t*_0_ (see Supporting Information S9 for *t*‐test statistics, *df*, and *p*‐values). In addition, females in Châteauvillain and Chizé differed in their relative growth rate. This provides evidence for sex and site differences in growth parameters for Gompertz type growth trajectory. These differences among parameters were not observed only for Gompertz trajectories, but also for logistic trajectories. Indeed, among individuals that displayed a body growth trajectory best described by a logistic model, we detected sex differences in relative growth rate *k* and site differences in both relative growth rate and timing of maximum growth *t_0_*. As monomolecular models provided a very poor fit for males in both sites, comparison among sexes for this specific growth shape was not possible. However, for females, we detected site differences in asymptotic body mass *A* (see Supporting Information S9). Overall, these findings provide strong statistical evidence of sex and site differences in both growth trajectories and growth parameters (for a given shape).

## 4 DISCUSSION

Thanks to multiple body mass measurements of wild boars throughout their lifetime in two populations exposed to markedly different ecological contexts, we found clear evidence for a high diversity of body growth trajectories, which are context‐, sex‐, and cohort‐specific.

### 4.1 Shape of growth trajectories

Based on previous knowledge on the association between the shape of body growth and the state of development at birth across mammals (Gaillard et al., 1997), as well as from previous modeling of pig growth (Ceron et al., 2020), we expected wild boar to exhibit a Gompertz type growth trajectory. Indeed, wild boar are closer to the altricial end of the altricial–precocial spectrum (following the criteria of Derrickson, 1992). Thus, the gestation period of wild boar is relatively short (ca 115 days; Henry, 1968) compared to similar‐sized ungulates such as bighorn sheep (*Ovis canadensis*, ca 174 days Shackleton et al., 1984) or ibex (*Capra ibex*, ca 167 days Stüwe & Grodinsky, 1987), leading to a shorter period for development in utero. In addition, piglets remain in nests during the first few days of life because they are born with a low amount of subcutaneous fat (Curtis & Rogler, 1970; Le Dividich & Noblet, 1983), and are thereby dependent on mothers for thermoregulation as well as food provisioning. In support, we found that the average body growth of wild boar displayed a Gompertz type trajectory in Châteauvillain, for both sexes. Individuals thus reach their maximum growth rate at about 37% of their adult mass. However, in Chizé, the average body growth of males displayed a logistic type trajectory, meaning that maximum growth rate is only reached when 50% of the asymptotic body mass has been reached, thus later than in Châteauvillain. For females, the monomolecular model performed best, indicating a consistently decreasing growth rate from first capture onwards. Individual‐specific growth trajectories, in accordance with average body growth at the population level, showed high individual heterogeneity in growth shape as well as growth metrics (i.e., *A*, *k,* and *t_0_*). Thus, wild boar does not always exhibit a Gompertz type growth trajectory. This finding may be explained by the ranking of wild boar along the altricial–precocial continuum. Indeed, wild boar may be considered as a partially precocial species (see Scheiber et al., 2017 for a review). They are born with their eyes open (sensory independence) and with teeth. Wild boar seems thus to be similar to spotted hyenas (*Crocuta crocuta*) in terms of the level of precocity at birth (Holekamp & Smale, 1998). Hyena exhibit a body growth following a monomolecular type characterized by an early timing of peak growth (Swanson et al., 2013) expected for precocial species (Gaillard et al., 1997). Our findings are thus partly consistent with the expected association between the shape of body growth and the state of development at birth across mammals. The high diversity of growth trajectories we report both within and between studied populations points out the unusual flexibility of body growth shape that characterizes wild boar, which is likely related to its intermediate ranking on the precocial‐altricial continuum of early development across mammalian species.

### 4.2 Sex‐specific body growth

Wild boars exhibit strong sexual size dimorphism in adulthood (Pedone et al., 1995; Toïgo et al., 2008). At birth, males are only slightly heavier than females (male fetuses have been recorded to be 5.1% heavier than females; Servanty et al., 2007; also see Gamelon et al., 2013, 2018). Wild boar body growth is not sex‐specific until at least six months (Gaillard et al., 1992; Veylit et al., 2020a) or even 1 year (Pépin, 1991) of age. Compared to other ungulate species, wild boars thus display similar early‐life growth trajectory between sexes, similar to monogamous and monomorphic mammalian species such as *Madoqua kirkii* (Jarman, 1983; Kellas, 1955), with both sexes growing at a similar rate. Sex‐specific differences in body growth rates therefore occur later in life (also see Pedone et al., 1995). Similar to domestic pigs, males grew at a faster rate than females mostly after maturity (Walstra, 1980), although the strength of this difference is a function of the environmental context. Differences in sex‐specific growth arise as females allocate resources to reproduction rather than growth when they reach their threshold mass for reproduction. Wild boar females at Châteauvillain (a heavily hunted and rich environment) displayed a low threshold mass for reproducing (about 27–33 kg corresponding to 33%–41% of adult body mass) compared to most other ungulate species (with a mass threshold of about 80% of adult body mass; Servanty et al., 2009; Gaillard et al., 2000), likely in response to the combined effect of abundant food resources and high hunting pressure. Interestingly, we found that females in Châteauvillain exhibited a Gompertz type trajectory, meaning that when 37% of the adult body mass is reached, growth rates tend to decrease, likely resulting from a diversion of resources from growth to reproduction. In Chizé, body growth rates in both sexes and asymptotic body masses are lower than in Châteauvillain due to resource limitation in this poorer environment as well as a weaker hunting pressure that does not select for reproducing as early as possible. According to the higher susceptibility of males than females to resource limitation in species under strong sexual selection (see, e.g., Leberg and Smith (1993) on white‐tailed deer, *Odocoileus virginianus*, Leblanc et al. (2001) on bighorn sheep *Ovis canadensis*), we expected sexual size dimorphism (SSD) to be lower in Chizé. In support to that expectation, the amount of SSD (computed as the log‐scaled ratio between the mean asymptotic adult body mass for males and the mean asymptotic adult body mass for females from the restricted dataset) was twice as high in Châteauvillain (SSD = 0.54) than in Chizé (SSD = 0.25) (see Supporting Information S10 for sex‐ and site‐specific distributions of asymptotic body masses).

### 4.3 Variation in adult body mass across cohorts

Adult body mass varied among cohorts in both sexes in both sites, indicating that early‐life conditions markedly influence adult body mass throughout the developmental process in wild boar. This finding has also been demonstrated experimentally in rats (McCance, 1962) and in a free‐ranging population of roe deer, *Capreolus capreolus* (Douhard et al., 2013). The long‐term benefits of favorable conditions at birth correspond to silver spoon effects (Grafen, 1988) and may generate strong cohort effects. Long‐term differences in performance between cohorts have been shown in a variety of taxa including birds (van der Jeugd & Larsson, 1998), fish (Wiegmann et al., 1997), and humans (Takei et al., 1996), to name just a few (see Tuljapurkar et al., 2021 for a review) and are often due to fluctuations in climatic conditions in natural populations (Post et al., 1997). In our study, the availability of food resources, mainly acorns, fluctuated within and across years (Gamelon et al., 2021; Touzot et al., 2020) in both sites. Fluctuating pulsed resources can indirectly influence growth from birth to weaning (at about 3 months of age) through temporal variation in milk quality provisioned to offspring (Gamelon et al., 2017; Yang et al., 2000) and can directly influence growth of piglets after weaning. The cohort effect we found on asymptotic body mass *A* in all sites and sexes is therefore consistent with the hypothesis that early‐life conditions play a key role in shaping adult body mass in wild boar.

### 4.4 Statistical evidence for site‐ and sex‐specific lifetime growth? A proof‐of‐concept

We investigated lifetime growth trajectories by fitting separate growth models to females at Chizé, females at Châteauvillain, males at Chizé, and males at Châteauvillain and found clear evidence for differences in the shape of growth. This heterogeneity prevented us to test directly for statistical differences in growth parameters between populations and sexes. For readers not convinced by the reasoning that different growth models means different growth patterns, we performed a formal quantitative comparison of sex and site effects using the whole dataset both under the assumption of a Gompertz and a logistic model, which were the most frequently selected models. The best Gompertz model fitted on the whole dataset (with a random intercept of individual identity on adult body mass *A*) testing for potential effects of site and sex on *A* led to retain the effects of sex and site (as categorical additive effects) as statistically significant, which indicates that the asymptotic body mass depends on both sex and site (Supporting Information S11). Similarly, the best Gompertz model testing for potential site and sex effects on *k* included sex and site as categorical additive effects that were statistically significant, which indicates that *k* depends on both sex and site (Supporting Information S11). Finally, the best Gompertz model testing for potential site and sex effects on *t*_0_ included sex and site as categorical interactive effects on *t*_0_ that were statistically significant, which indicates that *t*_0_ also depends on both sex and site (Supporting Information S11). The same best models were obtained for logistic models, providing a statistical support for site and sex effects on all growth parameters (*A*, *k,* and *t*_0_, see Supporting Information S11). Therefore, whatever the growth model we considered, growth parameters consistently differed between sexes and between sites. This analysis formally demonstrates the existence of statistically significant differences in sex‐ and site‐specific growth parameters in wild boar, for a given growth model (Gompertz or logistic). The combination of the approach used in this study, in which we fitted separate growth models to females at Chizé, females at Châteauvillain, males at Chizé and males at Châteauvillain, and this overall quantitative analysis, both clearly demonstrate a diversity of growth shapes and growth parameters among individuals of different sex from different sites.

## 5 CONCLUSIONS

Our findings provide evidence for a diversity of body growth trajectories in wild boar, which are shaped by the environmental context, the sex, and the year of birth. Observed site‐specific differences in body growth trajectories are likely related to the environmental context. Indeed, both the differing strength of hunting pressure and resource availability in the two study areas may have contributed to the differences in growth we report between populations. Females in Châteauvillain faced with a high hunting pressure that kept the population well below its carrying capacity, with a quite constant population growth rate (around 8% per year, Gamelon et al., 2012, 2021). This weak intra‐specific competition together with high food availability (mast seeding) allow females in this population to grow fast and become large. In contrast, females in Chizé are subject to a much weaker hunting pressure and have less food available due to the low percent cover by seed producing trees (Veylit et al., 2020b). Thus, in contrast to Châteauvillain, the population in Chizé is likely much closer to a demographic status of saturation. Potential density‐dependent effects should thus limit the body growth rate and asymptotic body mass of females in Chizé. In accordance with our predictions, males and females of this polygynous and dimorphic species differ in their growth trajectories, and sexual size dimorphism is stronger in the resource‐abundant environment. Finally, we found a high cohort variation in adult body mass, which suggests that environmental conditions early in life shape body mass at adulthood. Whether the intermediate ranking of this species on the precocial–altricial continuum may explain the ability of this species to exhibit various growth patterns has to be investigated in future studies. Exploring growth trajectories among and within populations for species with contrasting ranking along the precocial–altricial continuum thus offers promising avenues of research.

## CONFLICT OF INTEREST

None declared.

## AUTHOR CONTRIBUTIONS

**Lara Veylit:** Conceptualization (equal); Formal analysis (lead); Methodology (lead); Visualization (lead); Writing‐original draft (lead); Writing‐review & editing (equal). **Bernt‐Erik Sæther:** Funding acquisition (lead); Project administration (lead); Resources (lead); Supervision (supporting); Writing‐review & editing (equal). **Jean‐Michel Gaillard:** Conceptualization (equal); Investigation (equal); Methodology (equal); Supervision (supporting); Writing‐original draft (supporting); Writing‐review & editing (equal). **Eric Baubet:** Data curation (lead); Writing‐review & editing (equal). **Marlène Gamelon:** Conceptualization (equal); Formal analysis (equal); Methodology (equal); Supervision (lead); Visualization (supporting); Writing‐original draft (supporting); Writing‐review & editing (equal).

## Supporting information

Supplementary Material

## Data Availability

The data used in our analysis will be made available in Dryad upon publication of this study.

## References

[ece38164-bib-0001] Bertouille, S. B. , & de Crombrugghe, S. A. (1995). Body mass and lower jaw development of the female red deer as indices of habitat quality in the Ardennes. Acta Theriologica, 40, 145–162. 10.4098/at.arch.95-16

[ece38164-bib-0002] Brooks, M. E. , Clements, C. , Pemberton, J. , & Ozgul, A. (2017). Estimation of individual growth trajectories when repeated measures are missing. The American Naturalist, 190(3), 377–388. 10.1086/692797 28829634

[ece38164-bib-0003] Burnham, K. P. , & Anderson, D. R. (2002). Model selection and multimodel inference: A practical information‐theoretic approach (2nd ed.). Springer.

[ece38164-bib-0004] Case, T. J. (1978). On the evolution and adaptive significance of postnatal growth rates in the terrestrial vertebrates. The Quarterly Review of Biology, 53(3), 243–282. 10.1086/410622 362471

[ece38164-bib-0005] Ceron, M. S. , Oliveira, V. D. , Pieve, N. A. N. N. , Silva, N. C. D. E. , Rossi, C. A. R. , Fraga, B. N. , Muniz, H. D. C. M. , & Kessler, A. D. M. (2020). Nonlinear equations to determine the growth curve of immunocastrated pigs. Pesquisa Agropecuária Brasileira, 55. 10.1590/s1678-3921.pab2020.v55.01184

[ece38164-bib-0006] Chapman, N. G. , Furlong, M. , & Harris, S. (1997). Reproductive strategies and the influence of date of birth on growth and sexual development of an aseasonally‐breeding ungulate: Reeves' muntjac (*Muntiacus reevesi*). Journal of Zoology, 241(3), 551–570.

[ece38164-bib-0007] Cody, M. L. (1966). A general theory of clutch size. Evolution, 174–184. 10.1111/j.1558-5646.1966.tb03353.x 28563630

[ece38164-bib-0008] Coulson, T. (2012). The Per Brinck Oikos Award 2012 ‐ Tim Coulson. Oikos, 121, 1. 10.1111/j.1600-0706.2011.20361.x

[ece38164-bib-0009] Curtis, S. E. , & Rogler, J. C. (1970). Thermoregulatory ontogeny in piglets: Sympathetic and adipokinetic responses to cold. American Journal of Physiology‐Legacy Content, 218(1), 149–152. 10.1152/ajplegacy.1970.218.1.149 5410068

[ece38164-bib-0010] Derrickson, E. M. (1992). Comparative reproductive strategies of altricial and precocial eutherian mammals. Functional Ecology, 6(1), 57–65. 10.2307/2389771

[ece38164-bib-0011] Douhard, M. , Gaillard, J.‐M. , Delorme, D. , Capron, G. , Duncan, P. , Klein, F. , & Bonenfant, C. (2013). Variation in adult body mass of roe deer: Early environmental conditions influence early and late body growth of females. Ecology, 94(8), 1805–1814. 10.1890/13-0034.1 24015524

[ece38164-bib-0012] English, S. , Bateman, A. W. , & Clutton‐Brock, T. H. (2012). Lifetime growth in wild meerkats: Incorporating life history and environmental factors into a standard growth model. Oecologia, 169(1), 143–153. 10.1007/s00442-011-2192-9 22108854

[ece38164-bib-0013] Festa‐Bianchet, M. , Douhard, M. , Gaillard, J. M. , & Pelletier, F. (2017). Successes and challenges of long‐term field studies of marked ungulates. Journal of Mammalogy, 98(3), 612–620. 10.1093/jmammal/gyw227

[ece38164-bib-0014] Festa‐Bianchet, M. , King, W. J. , Jorgenson, J. T. , Smith, K. G. , & Wishart, W. D. (1996). The development of sexual dimorphism: Seasonal and lifetime mass changes in bighorn sheep. Canadian Journal of Zoology, 74, 330–342. 10.1139/z96-041

[ece38164-bib-0015] Gaillard, J.‐M. , Duncan, P. , Delorme, D. , Van Laere, G. , Pettorelli, N. , Maillard, D. , & Renaud, G. (2003). Effects of Hurricane Lothar on the population dynamics of European Roe Deer. The Journal of Wildlife Management, 67, 767. 10.2307/3802684

[ece38164-bib-0016] Gaillard, J. M. , Festa‐Bianchet, M. , Delorme, D. , & Jorgenson, J. (2000). Body mass and individual fitness in female ungulates: Bigger is not always better. Proceedings. Biological Sciences, 267(1442), 471–477. 10737404 10.1098/rspb.2000.1024 PMC1690550

[ece38164-bib-0017] Gaillard, J. M. , Pontier, D. , Allaine, D. , Loison, A. , Herve, J. C. , & Heizmann, A. (1997). Variation in growth form and precocity at birth in eutherian mammals. Proceedings. Biological Sciences, 264(1383), 859–868. 9225478 10.1098/rspb.1997.0120 PMC1688443

[ece38164-bib-0084] Gaillard, J. M. , Brandt, S. , & Jullien, J. M. (1993). Body weight effect on reproduction of young wild boar (*Sus scrofa*) females: A comparative analysis. Folia Zoologica, 42(3), 204–212.

[ece38164-bib-0018] Gaillard, J.‐M. , Pontier, D. , Brandt, S. , Jullien, J.‐M. , & Allainé, D. (1992). Sex differentiation in postnatal growth rate: A test in a wild boar population. Oecologia, 90, 167–171. 10.1007/bf00317173 28313711

[ece38164-bib-0019] Gallo Orsi, U. , Macchi, E. , Perrone, A. , & Durio, P. (1995). Biometric data and growth rates of wild boar population living in the Italian Alps. IBEX – Journal of Mountain Ecology, 3, 60–63.

[ece38164-bib-0020] Gamelon, M. , Besnard, A. , Gaillard, J.‐M. , Servanty, S. , Baubet, E. , Brandt, S. , & Gimenez, O. (2011). High hunting pressure selects for earlier birth date: Wild boar as a case study. Evolution; International Journal of Organic Evolution, 65(11), 3100–3112. 10.1111/j.1558-5646.2011.01366.x 22023578

[ece38164-bib-0021] Gamelon, M. , Focardi, S. , Baubet, E. , Brandt, S. , Franzetti, B. , Ronchi, F. , Venner, S. , Sæther, B. E. , & Gaillard, J. M. (2017). Reproductive allocation in pulsed‐resource environments: A comparative study in two populations of wild boar. Oecologia, 183(4), 1065–1076. 10.1007/s00442-017-3821-8 28154966

[ece38164-bib-0022] Gamelon, M. , Gaillard, J. M. , Baubet, E. , Devillard, S. , Say, L. , Brandt, S. , & Gimenez, O. (2013). The relationship between phenotypic variation among offspring and mother body mass in wild boar: Evidence of coin‐flipping? Journal of Animal Ecology, 82(5), 937–945. 10.1111/1365-2656.12073 23495696

[ece38164-bib-0023] Gamelon, M. , Gayet, T. , Baubet, E. , Devillard, S. , Say, L. , Brandt, S. , Pélabon, C. , & Sæther, B. E. (2018). Does multiple paternity explain phenotypic variation among offspring in wild boar? Behavioral Ecology, 29(4), 904–909.

[ece38164-bib-0024] Gamelon, M. , Touzot, L. , Baubet, E. , Cachelou, J. , Focardi, S. , Franzetti, B. , Nivois, E. , Veylit, L. , & Sæther, B. E. (2021). Effects of pulsed resources on the dynamics of seed consumer populations: A comparative demographic study in wild boar. Ecosphere, 12(5), e03395. 10.1002/ecs2.3395

[ece38164-bib-0025] Garel, M. , Solberg, E. J. , Saether, B.‐E. , Herfindal, I. , & Høgda, K.‐A. (2006). The length of growing season and adult sex ratio affect sexual size dimorphism in moose. Ecology, 87(3), 745–758. 10.1890/05-0584 16602303

[ece38164-bib-0026] Gélin, U. , Wilson, M. E. , Cripps, J. , Coulson, G. , & Festa‐Bianchet, M. (2016). Individual heterogeneity and offspring sex affect the growth–reproduction trade‐off in a mammal with indeterminate growth. Oecologia, 180(4), 1127–1135. 10.1007/s00442-015-3531-z 26714827

[ece38164-bib-0027] Grafen, A. (1988). On the uses of data on lifetime reproductive success. In T. Clutton‐Brock (Ed.), Reproductive success studies of individual variation in contrasting breeding systems, Chapter 28 (pp. 454–471). University of Chicago Press.

[ece38164-bib-0028] Green, W. C. H. , & Rothstein, A. (1991). Trade‐offs between growth and reproduction in female bison. Oecologia, 86(4), 521–527. 10.1007/BF00318318 28313333

[ece38164-bib-0029] Hafiz, M. , Izuan Bahtiar, A. W. , Mohamad Hifzan, A. J. , Ariff, O. M. , & Faezal Ashraff, A. L. (2016). Describing growth pattern of Bali cows using non‐linear regression models. Malaysian Society of Animal Production, 19(2), 1–7.

[ece38164-bib-0030] Henry, V. G. (1968). Length of estrous cycle and gestation in European wild hogs. The Journal of Wildlife Management, 32(2), 406–408. 10.2307/3798986

[ece38164-bib-0031] Holekamp, K. E. , & Smale, L. (1998). Behavioral development in the spotted hyena. BioScience, 48(12), 997–1005. 10.2307/1313456

[ece38164-bib-0032] Howells, W. W. , & Hanks, J. (1976). Body growth of the impala (*Aepyceros mefampus*) in Wankie National Park, Rhodesia. South African Journal of Wildlife Research, 5(2), 95–98.

[ece38164-bib-0033] Jarman, P. (1983). Mating system and sexual dimorphism in large terrestrial mammalian herbivores. Biological Reviews, 58, 485–520. 10.1111/j.1469-185x.1983.tb00398.x

[ece38164-bib-0034] Kellas, L. (1955). Observations on the reproductive activities, measurements, and growth rate of the dik‐dik (*Rhynchotragus kirkii thomasi Neumann*). Proceedings of the Zoological Society of London, 124(75), 1–784.

[ece38164-bib-0035] Knott, K. K. , Barboza, P. S. , & Terry Bowyer, R. (2005). Growth in arctic ungulates: Postnatal development and organ maturation in *Rangifer tarandus* and *Ovibos moschatus* . Journal of Mammalogy, 86, 121–130.

[ece38164-bib-0036] Kor, A. , Baspinar, E. , Karaca, S. , & Keskin, S. (2011). The determination of growth in Akkeci (White goat) female kids by various growth models. Czech Journal of Animal Science, 51, 110–116. 10.17221/3917-cjas

[ece38164-bib-0037] Le Dividich, J. , & Noblet, J. (1983). Thermoregulation and energy metabolism in the neonatal pig. Annales de Recherches Veterinaires, 14(4), 375–381. 6677178

[ece38164-bib-0038] Leberg, P. L. , Brisbin, I. L. , Smith, M. H. , & White, G. C. (1989). Factors affecting the analysis of growth patterns of large mammals. Journal of Mammalogy, 70, 275–283. 10.2307/1381508

[ece38164-bib-0039] Leberg, P. L. , & Smith, M. H. (1993). Influence of density on growth of white‐tailed deer. Journal of Mammalogy, 74, 723–731. 10.2307/1382294

[ece38164-bib-0040] LeBlanc, M. , Festa‐Bianchet, M. , & Jorgenson, J. T. (2001). Sexual size dimorphism in bighorn sheep (*Ovis canadensis*): Effects of population density. Canadian Journal of Zoology, 79(9), 1661–1670.

[ece38164-bib-0041] Marcil‐Ferland, D. , Festa‐Bianchet, M. , Martin, A. M. , & Pelletier, F. (2013). Despite catch‐up, prolonged growth has detrimental fitness consequences in a long‐lived vertebrate. The American Naturalist, 182, 775–785. 10.1086/673534 24231538

[ece38164-bib-0042] McCance, R. A. (1962). Food, growth, and time. The Lancet, 280(7257), 621–626. 10.1016/S0140-6736(62)92539-4 13932033

[ece38164-bib-0043] Mitchell, B. , Staines, B. W. , & Welch, B. (1977). Ecology of Red Deer‐A research review relevant to their management in Scotland. Institute of Terrestrial Ecology.

[ece38164-bib-0044] Monteith, K. L. , Schmitz, L. E. , Jenks, J. A. , Delger, J. A. , & Terry Bowyer, R. (2009). Growth of Male White‐Tailed Deer: Consequences of maternal effects. Journal of Mammalogy, 90, 651–660. 10.1644/08-mamm-a-191r1.1

[ece38164-bib-0045] Pedone, P. , Mattioli, S. , & Mattioli, L. (1995). Body size and growth patterns in wild boars of Tuscany, Central Italy. Journal of Mountain Ecology, 3, 66–68.

[ece38164-bib-0046] Pépin, D. (1991). Alimentation,croissance et reproduction chez la laie :études en conditions naturelles et en captivité. INRAE Productions Animales, 4(2), 183–189.

[ece38164-bib-0047] Pettorelli, N. , Gaillard, J.‐M. , Mysterud, A. , Duncan, P. , Chr. Stenseth, N. , Delorme, D. , Van Laere, G. , Toïgo, C. , & Klein, F. (2006). Using a proxy of plant productivity (NDVI) to find key periods for animal performance: The case of roe deer. Oikos, 112, 565–572. 10.1111/j.0030-1299.2006.14447.x

[ece38164-bib-0048] Pinheiro, J. , Bates, D. , DebRoy, S. , Sarkar, D. , R Core Team (2020). nlme: Linear and Nonlinear Mixed Effects Models. R package version 3.1‐149. https://CRAN.R‐project.org/package=nlme

[ece38164-bib-0049] Plard, F. , Gaillard, J. M. , Coulson, T. , Delorme, D. , Warnant, C. , Michallet, J. , Tuljapurkar, S. , Krishnakumar, S. , & Bonenfant, C. (2015). Quantifying the influence of measured and unmeasured individual differences on demography. Journal of Animal Ecology, 84(5), 1434–1445. 10.1111/1365-2656.12393 PMC5642278 26140296

[ece38164-bib-0050] Post, E. , Stenseth, N. C. , Langvatn, R. , & Fromentin, J. M. (1997). Global climate change and phenotypic variation among red deer cohorts. Proceedings of the Royal Society of London. Series B: Biological Sciences, 264(1386), 1317–1324. 9332016 10.1098/rspb.1997.0182 PMC1688584

[ece38164-bib-0051] R Development Core Team (2020). R: A language and environment for statistical computing. R Foundation for Statistical Computing.

[ece38164-bib-0052] Reiss, M. J. (1989). The allometry of growth and reproduction. Cambridge University Press.

[ece38164-bib-0053] Robinette, W. L. , & Child, G. F. T. (1964). Notes on biology of the lechwe (*Kobus leche*). The Puku, 2, 84–117.

[ece38164-bib-0054] Sand, H. , Cederlund, G. , & Danell, K. (1995). Geographical and latitudinal variation in growth patterns and adult body size of Swedish moose (*Alces alces*). Oecologia, 102(4), 433–442. 10.1007/BF00341355 28306886

[ece38164-bib-0086] Scheiber, I. B. , Weiß, B. M. , Kingma, S. A. , & Komdeur, J. (2017). The importance of the altricial–precocial spectrum for social complexity in mammals and birds–A review. Frontiers in Zoology, 14(1), 1–20. 28115975 10.1186/s12983-016-0185-6 PMC5242088

[ece38164-bib-0055] Servanty, S. , Gaillard, J. M. , Allainé, D. , Brandt, S. , & Baubet, E. (2007). Litter size and fetal sex ratio adjustment in a highly polytocous species: The wild boar. Behavioral Ecology, 18(2), 427–432. 10.1093/beheco/arl099

[ece38164-bib-0056] Servanty, S. , Gaillard, J. M. , Toïgo, C. , Serge, B. , & Baubet, E. (2009). Pulsed resources and climate‐induced variation in the reproductive traits of wild boar under high hunting pressure. The Journal of Animal Ecology, 78(6), 1278–1290. 10.1111/j.1365-2656.2009.01579.x 19549145

[ece38164-bib-0085] Servanty, S. , Gaillard, J. M. , Ronchi, F. , Focardi, S. , Baubet, E. , & Gimenez, O. (2011). Influence of harvesting pressure on demographic tactics: Implications for wildlife management. Journal of Applied Ecology, 48(4), 835–843.

[ece38164-bib-0057] Shackleton, D. M. , Peterson, R. G. , Haywood, J. , & Bottrell, A. (1984). Gestation period in *Ovis canadensis* . Journal of Mammalogy, 65(2), 337–338. 10.2307/1381176

[ece38164-bib-0058] Sinclair, A. R. E. (1977). The African Buffalo: A study of resource limitation of populations. The University of Chicago Press.

[ece38164-bib-0059] Solberg, E. J. , Garel, M. , Heim, M. , Grøtan, V. , & Sæther, B.‐E. (2008). Lack of compensatory body growth in a high performance moose *Alces alces* population. Oecologia, 158, 485–498. 10.1007/s00442-008-1158-z 18830632

[ece38164-bib-0060] Spinage, C. A. (1969). Waterbuck management data. East African Agriculture and Forestry Journal, 34(3), 327–335. 10.1080/00128325.1969.11662312

[ece38164-bib-0061] Stearns, S. C. (1992). The evolution of life histories. Oxford University Press on Demand.

[ece38164-bib-0062] Strathe, A. B. , Danfaer, A. , Chwalibog, A. , Sørensen, H. , & Kebreab, E. (2010). A multivariate nonlinear mixed effects method for analyzing energy partitioning in growing pigs. Journal of Animal Science, 88(7), 2361–2372. 20348377 10.2527/jas.2009-2065

[ece38164-bib-0063] Stüwe, M. , & Grodinsky, C. (1987). Reproductive biology of captive Alpine ibex (*Capra i. ibex*). Zoo Biology, 6(4), 331–339. 10.1002/zoo.1430060407

[ece38164-bib-0064] Suzuki, M. , Onuma, M. , Yokoyama, M. , Kaji, K. , Yamanaka, M. , & Ohtaishi, N. (2001). Body size, sexual dimorphism, and seasonal mass fluctuations in a larger sika deer subspecies, the Hokkaido sika deer (*Cervus nippon yesoensis* Heude, 1884). Canadian Journal of Zoology, 79(1), 154–159.

[ece38164-bib-0065] Swanson, E. M. , McElhinny, T. L. , Dworkin, I. , Weldele, M. L. , Glickman, S. E. , & Holekamp, K. E. (2013). Ontogeny of sexual size dimorphism in the spotted hyena (*Crocuta crocuta*). Journal of Mammalogy, 94(6), 1298–1310.

[ece38164-bib-0066] Takei, N. , Lewis, G. , Sham, P. C. , & Murray, R. M. (1996). Age‐period‐cohort analysis of the incidence of schizophrenia in Scotland. Psychological Medicine, 26, 963–973. 10.1017/S0033291700035297 8878329

[ece38164-bib-0067] Tiilikainen, R. , Nygrén, T. , Pusenius, J. , & Ruusila, V. (2010). Variation in growth pattern of male moose Alces alces after two contrasted periods of hunting. Annales Zoologici Fennici, 47(3), 159–172.

[ece38164-bib-0068] Toïgo, C. , Servanty, S. , Gaillard, J. M. , Brandt, S. , & Baubet, E. (2008). Disentangling natural from hunting mortality in an intensively hunted wild boar population. The Journal of Wildlife Management, 72(7), 1532–1539.

[ece38164-bib-0069] Touzot, L. , Schermer, É. , Venner, S. , Delzon, S. , Rousset, C. , Baubet, É. , Gaillard, J. M. , & Gamelon, M. (2020). How does increasing mast seeding frequency affect population dynamics of seed consumers? Wild boar as a case study. Ecological Applications, 30(6), e02134. 10.1002/eap.2134 32299142

[ece38164-bib-0070] Traill, L. W. , Schindler, S. , & Coulson, T. (2014). Demography, not inheritance, drives phenotypic change in hunted bighorn sheep. Proceedings of the National Academy of Sciences of the United States of America, 111(36), 13223–13228. 10.1073/pnas.1407508111 25114219 PMC4246946

[ece38164-bib-0071] Tuljapurkar, S. , Zuo, W. , Coulson, T. , Horvitz, C. , & Gaillard, J. M. (2021). Distributions of LRS in varying environments. Ecology Letters, 24(7), 1328–1340. 10.1111/ele.13745 33904254

[ece38164-bib-0072] van der Jeugd, H. P. , & Larsson, K. (1998). Pre‐breeding survival of barnacle geese *Branta leucopsis* in relation to fledgling characteristics. Journal of Animal Ecology, 67(6), 953–966. 10.1046/j.1365-2656.1998.6760953.x 26412374

[ece38164-bib-0073] van Noordwijk, A. , & de Jong, G. (1986). Acquisition and allocation of resources: Their influence on variation in life history tactics. The American Naturalist, 128(1), 137–142. 10.1086/284547

[ece38164-bib-0074] Veylit, L. , Sæther, B.‐E. , Gaillard, J.‐M. , Baubet, E. , & Gamelon, M. (2020a). Grow fast at no cost: No evidence for a mortality cost for fast early‐life growth in a hunted wild boar population. Oecologia, 192(4), 999–1012. 10.1007/s00442-020-04633-9 32242324 PMC7165149

[ece38164-bib-0075] Veylit, L. , Sæther, B. , Gaillard, J. , Baubet, E. , & Gamelon, M. (2020b). How do conditions at birth influence early‐life growth rates in wild boar? Ecosphere, 11, 3167. 10.1002/ecs2.3167

[ece38164-bib-0076] Vincek, D. , Sabo, K. , Kušec, G. , Kralik, G. , Đurkin, I. , & Scitovski, R. (2012). Modeling of pig growth by S‐function–least absolute deviation approach for parameter estimation. Archives Animal Breeding, 55(4), 364–374. 10.5194/aab-55-364-2012

[ece38164-bib-0077] Walstra, P. (1980). Growth and carcass composition from birth to maturity in relation to feeding level and sex in Dutch Landrace pigs.

[ece38164-bib-0078] Wellock, I. J. , Emmans, G. C. , & Kyriazakis, I. (2004). Describing and predicting potential growth in the pig. Animal Science, 78(3), 379–388. 10.1017/S1357729800058781

[ece38164-bib-0079] Wiegmann, D. D. , Baylis, J. R. , & Hoff, M. H. (1997). Male fitness, body size and timing of reproduction in smallmouth bass, *Micropterus dolomieui* . Ecology, 78(1), 111–128.

[ece38164-bib-0080] Wolverton, S. , Huston, M. A. , Kennedy, J. H. , Cagle, K. , & Cornelius, J. D. (2009). Conformation to Bergmann’s Rule in white‐tailed deer can be explained by food availability. The American Midland Naturalist, 162, 403–417. 10.1674/0003-0031-162.2.403

[ece38164-bib-0081] Yang, H. , Pettigrew, J. E. , Johnston, L. J. , Shurson, G. C. , & Walker, R. D. (2000). Lactational and subsequent reproductive responses of lactating sows to dietary lysine (protein) concentration. Journal of Animal Science, 78, 348. 10.2527/2000.782348x 10709925

[ece38164-bib-0082] Zöttl, M. , Vullioud, P. , Mendonça, R. , Torrents Ticó, M. , Gaynor, D. , Mitchell, A. , & Clutton‐Brock, T. (2016). Differences in cooperative behavior among Damaraland mole rats are consequences of an age‐related polyethism. Proceedings of the National Academy of Sciences of the United States of America, 113(37), 10382–10387. 10.1073/pnas.1607885113 27588902 PMC5027444

[ece38164-bib-0083] Zullinger, E. M. , Ricklefs, R. E. , Redford, K. H. , & Mace, G. M. (1984). Fitting sigmoidal equations to mammalian growth curves. Journal of Mammalogy, 65, 607–636. 10.2307/1380844

