# Supplementary material for: Many lifetime growth trajectories for a single mammal
Source: Ecol Evol. 2021 Oct 1;11(21):14789–804. doi: 10.1002/ece3.8164 (PMC8571586; doi:10.1002/ece3.8164)
Supplement: Supplementary file 1 — Supplementary Material [file ECE3-11-14789-s001.docx]

**Supporting Information for the article**

**“Many lifetime growth trajectories for a single mammal”**

**by**

Lara Veylit, Bernt-Erik Sæther, Jean-Michel Gaillard, Eric Baubet, and Marlène Gamelon


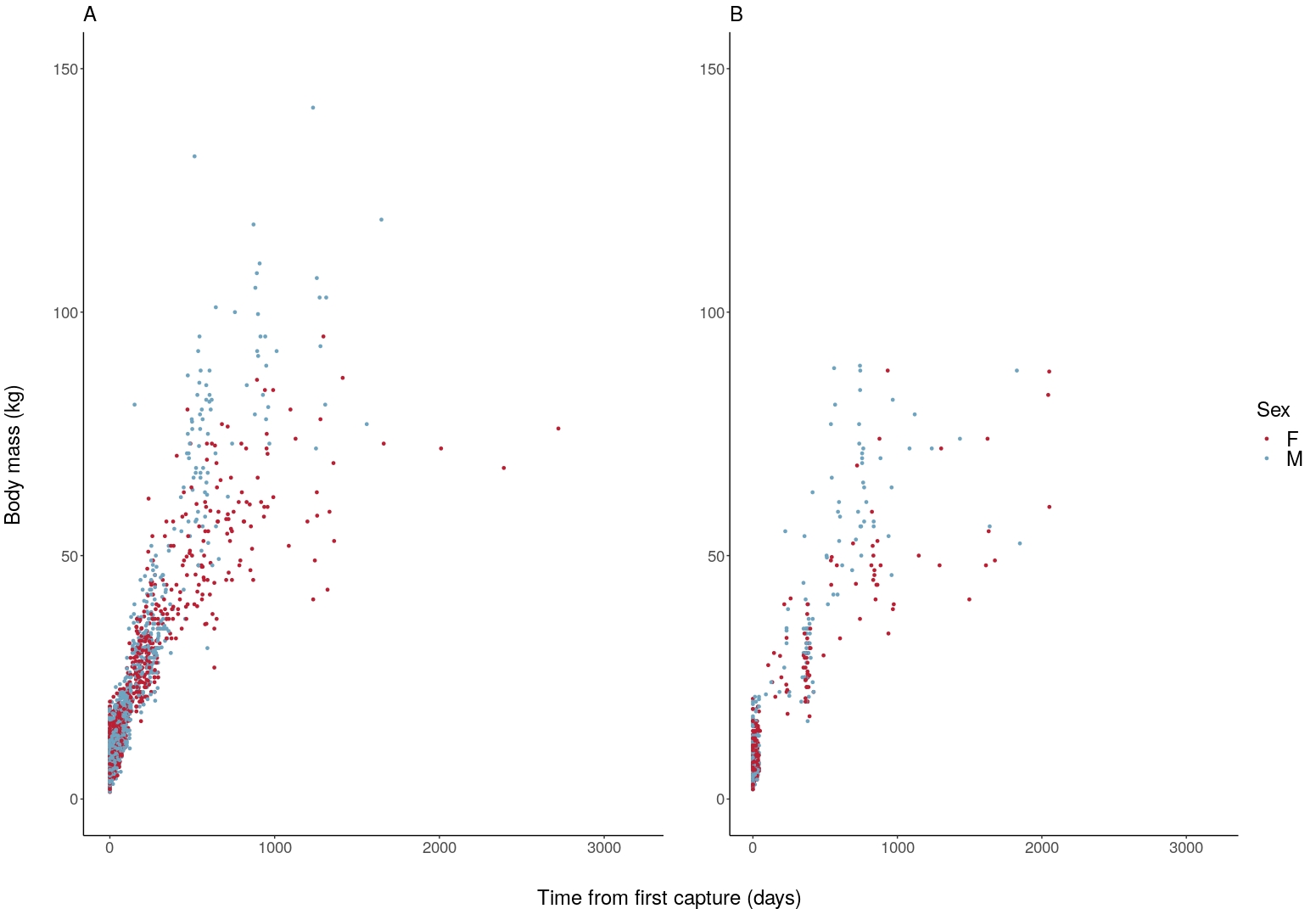


**Figure S1:** Time from first capture in days for each body mass measurement for wild boar in Châteauvillain (A) and Chizé (B), France. Data is from the full dataset for males (shown with blue points), and females (red points). These plots depict all mass measurements for 465 individuals (248 males, 217 females) at Châteauvillain and 156 individuals (83 males, 73 females) at Chizé included in the analyses.


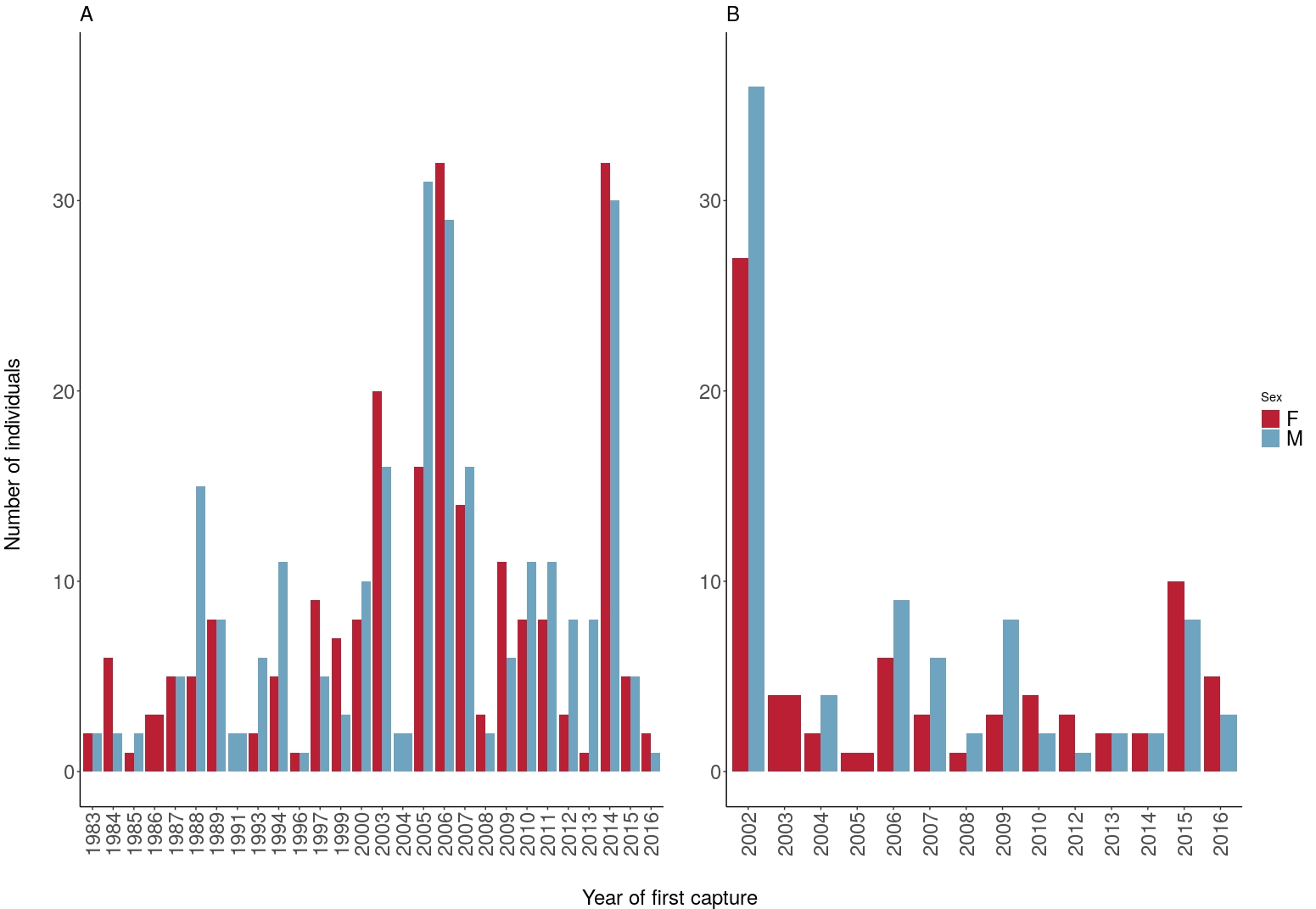


**Figure S2:** Year of first capture (i.e. birth year) for wild boar in Châteauvillain (A) and Chizé (B), France. Data for females is given in red, for males in blue.


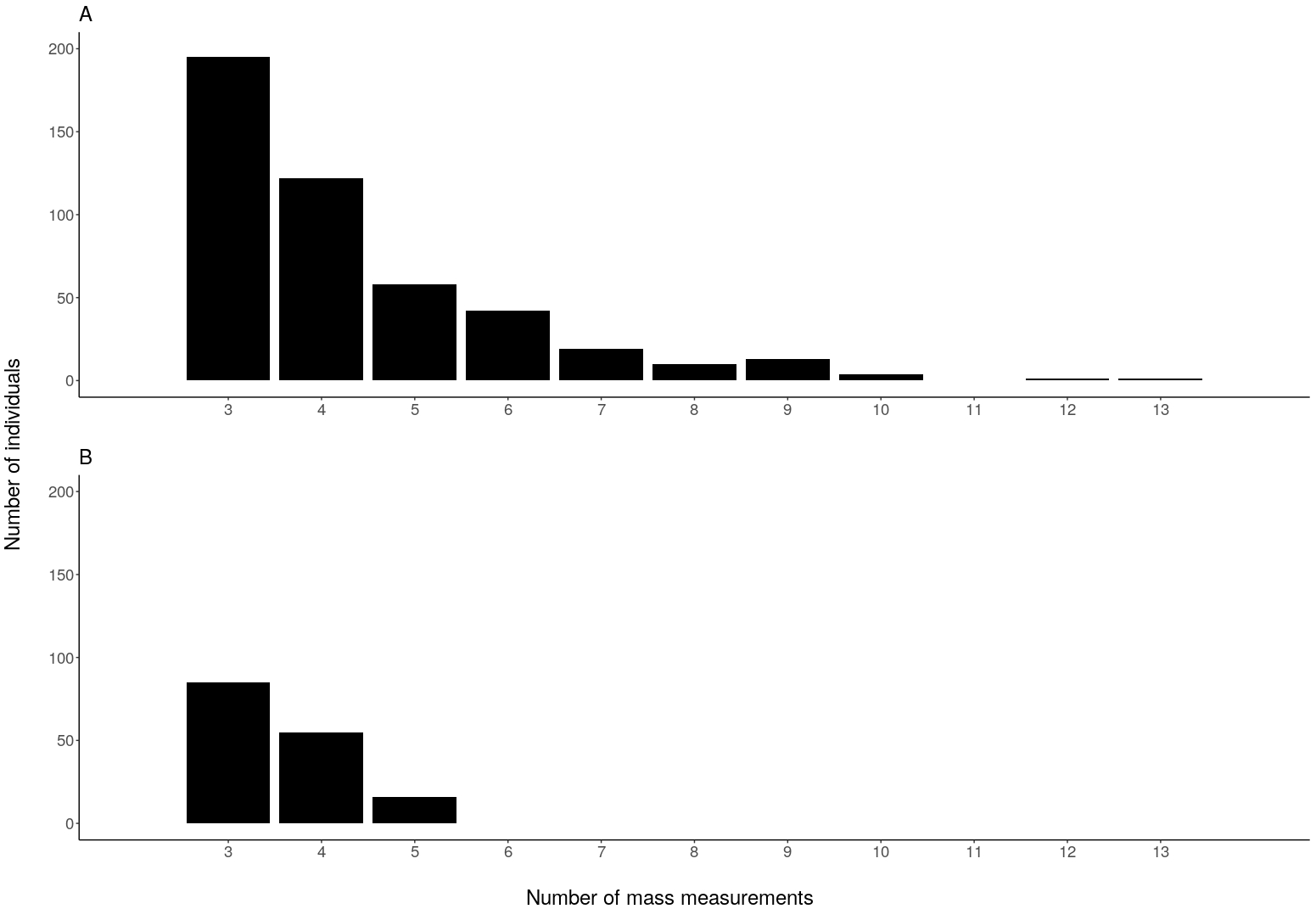


**Figure S3**: Number of mass measurements (from capture and recovery data) for wild boar at Châteauvillain (A) and Chizé (B), France. The distributions are for the full datasets.

**Supplementary Information S4:** R code used to implement the analyses (examples are for male wild boar at Châteauvillain).

############ Examples of models for Table 2 ############

#Variables included are

#Weight = weight at capture (in g)

#age_dfc = days from first capture date for each measurement

#ID = unique identification number for each individual

#A, k, t0 are defined in Table 2 per model

#birth.year = a given individual’s year of birth (as a factor)

library(nlme)

library(tidyverse)

#Gompertz

#No random effects, no fixed effects

gompertz.M <- nls(Weight ~ SSgompertz(age_dfc, A, k, t0) , data=M)

#Random effect A

gompertz.M.A=nlme(Weight~A*(exp(-exp(-k*(age_dfc-t0)))),

fixed=A+k+t0~1, random= 1/A~1|ID, data=M,

method = "ML",

start=coef(gompertz.M), na.action=na.omit)

#Random effect t0

gompertz.M.t0=nlme(Weight~A*(exp(-exp(-k*(age_dfc-t0)))),

fixed=A+k+t0~1, random= 1/t0~1|ID, data=M

method = "ML",

start=coef(gompertz.M), na.action=na.omit)

#Random effect k

gompertz.M.k=nlme(Weight~A*(exp(-exp(-k*(age_dfc-t0)))),

fixed=A+k+t0~1, random= 1/k~1|ID, data=M,

start=coef(gompertz.M), na.action=na.omit)

#Random effect A, birth year categorical on A

gompertz.M.A.BY =nlme(Weight~A*(exp(-exp(-k*(age_dfc-t0)))),

fixed=list(A ~ birth.year, k + t0 ~ 1), random= 1/A~1|ID,

data=M, start=list(fixed= c(A=c(rep(920000, 27)), k=2.25, t0=0.32)),

na.action=na.omit)

#Random effect t0, birth year categorical on A

gompertz.M.t0.BY =nlme(Weight~A*(exp(-exp(-k*(age_dfc-t0)))),

fixed=list(A ~ birth.year, k + t0 ~ 1), random= 1/t0~1|ID,

data=M, start=list(fixed= c(A=c(rep(920000, 27)), k=2.25, t0=0.32)),

na.action=na.omit)

#Random effect k, birth year categorical on A

gompertz.M.k.BY =nlme(Weight~A*(exp(-exp(-k*(age_dfc-t0)))),

fixed=list(A ~ birth.year, k + t0 ~ 1), random= 1/k~1|ID,

data=M, start=list(fixed= c(A=c(rep(920000, 27)), k=2.25, t0=0.32)),

na.action=na.omit)

#Logistic model, no random effects, no fixed effects

logit.M <- nls(Weight ~ SSlogis(age_dfc, A, k, t0) , data=M)

#Random effect A

logit.M.A <-nlme(Weight~A/(1+exp(-k*(age_dfc-t0))),

fixed=A+k+t0~1, random=1/A~1|ID, data=M,

start=coef(logit.M), na.action=na.omit)

#Random effect t0

logit.M.t0 <-nlme(Weight~A/(1+exp(-k*(age_dfc-t0))),

fixed=A+k+t0~1, random=1/t0~1|ID, data=M,

start=coef(logit.M), na.action=na.omit)

#Random effect k

logit.M.k <-nlme(Weight~A/(1+exp(-k*(age_dfc-t0))),

fixed=A+k+t0~1, random=1/k~1|ID, data=M,

start=coef(logit.M), na.action=na.omit)

#Random effect A, birth year categorical on A

logit.M.A.by = nlme(Weight~A/(1+exp(-k*(age_dfc-t0))),

fixed=list(A ~ birth.year, k + t0 ~ 1), random= 1/A~1|ID,

data=M, start=list(fixed= c(A=c(rep(920000, 27)), k=2.25, t0=0.32)),

na.action=na.omit)

#Random effect t0, birth year categorical on A

logit.M.t0.by = nlme(Weight~A/(1+exp(-k*(age_dfc-t0))),

fixed=list(A ~ birth.year, k + t0 ~ 1),random= 1/t0~1|ID,

data=M, start=list(fixed= c(A=c(rep(920000, 27)), k=2.25, t0=0.32)),

na.action=na.omit)

#Random effect k, birth year categorical on A

logit.M.k.by =nlme(Weight~A/(1+exp(-k*(age_dfc-t0))),

fixed=list(A ~ birth.year, k + t0 ~ 1), random= 1/k~1|ID,

data=M, start=list(fixed= c(A=c(rep(920000, 27)), k=2.25, t0=0.32)),

na.action=na.omit)

#Monomolecular no fixed effects, no random effects

Asymp.B.M <- nls(Weight ~ SSasymp(age_dfc, A, t0, k) , data=M)

#A random effect

Asymp.B.M.A<- nlme(Weight~ SSasymp(age_dfc, A, t0, k),

fixed=A+k+t0~1, random = 1/A~1|ID, data=M,

start=coef(Asymp.B.M ), na.action=na.omit)

# t0 random effect

Asymp.B.M.t0<- nlme(Weight~ SSasymp(age_dfc, A, t0, k),

fixed=A+k+t0~1, random = 1/t0~1|ID, data=M,

start=coef(Asymp.B.M ), na.action=na.omit)

# k random effect

Asymp.B.M.k<- nlme(Weight~ SSasymp(age_dfc, A, t0, k),

fixed=A+k+t0~1, random = 1/k~1|ID, data=M,

start=coef(Asymp.B.M ), na.action=na.omit)

#Random effect A, birth year categorical on A

Asymp.B.M.A.BY <- nlme(Weight~ SSasymp(age_dfc, A, t0, k),

fixed=list(A ~ birth.year, k+t0 ~ 1),

random = 1/A~1|ID, data=M,

start=list(fixed= c(A=c(rep(100000, 12)), k=0.32, t0=-0.980)),

na.action=na.omit)

#Random effect t0, birth year categorical on A

Asymp.B.M.t0.BY <- nlme(Weight~ SSasymp(age_dfc, A, k, t0),

fixed=list(A ~ birth.year, k+t0 ~ 1),

random = 1/t0~1|ID, data=M,

start=list(fixed= c(A=c(rep(100000, 12)), k=0.32, t0=-0.980)),

na.action=na.omit)

#Random effect k, birth year categorical on A

Asymp.B.M.k.BY <- nlme(Weight~ SSasymp(age_dfc, A, k, t0),

fixed=list(A ~ birth.year, k+t0 ~ 1),

random = 1/k~1|ID, data=M,

start=list(fixed= c(A=c(rep(100000, 12)), k=0.32, t0=-0.980)),

na.action=na.omit)

############ Examples of models for Table 3 ############

# This example is for storing the coefficients for individually-fit models. Models were fit for males with the multiple later life measurements.

for (i in 1:8)

{data1<-subset(M, M$ID==ident[i])

gompertz.M <- nls(Weight ~ SSgompertz(age_dfc, A, k, t0) , data=data1)

 gompertz<-nls(Weight~ A*(exp(-exp(-k*(age_dfc-t0)))) , start=coef(gompertz.M), data=data1)

 logistic<-nls(Weight~A/(1+exp(-k*(age_dfc-t0))), start = list(A=60000,k=2.1,t0=0.3), data=data1)

mono <- nls(Weight ~ SSasymp(age_dfc, A, t0, k) , data=data1)

}

**Figure S5:** Body growth trajectories (monomolecular, Gompertz and logistic) and associated AIC for males at Châteauvillain, from the dataset restricted to individuals with repeated measurements during both early and late in life. Each plot depicts the three models fit to a given individual’s data with points depicting per-individual observations.


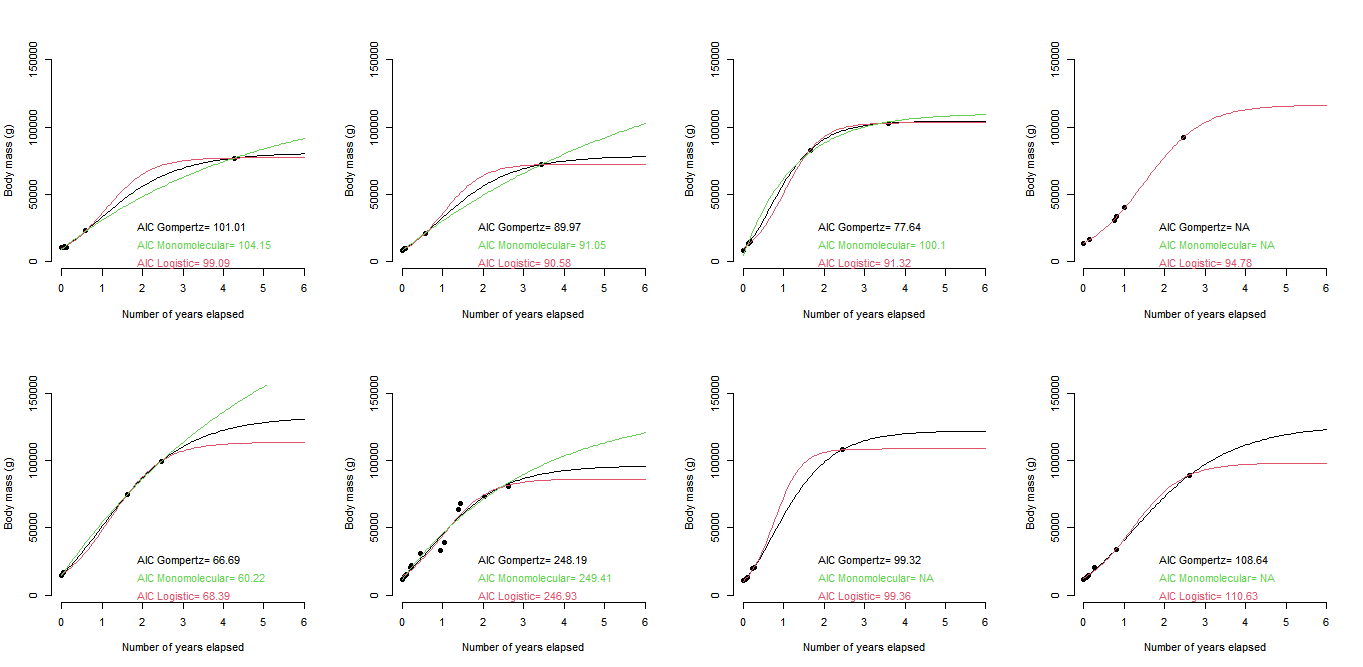


**Figure S6:** Body growth trajectories (monomolecular, Gompertz and logistic) and associated AIC for females at Châteauvillain, from the dataset restricted to individuals with repeated measurements during both early and late in life. Each plot depicts the three models fit to a given individual’s data with points depicting per-individual observations.


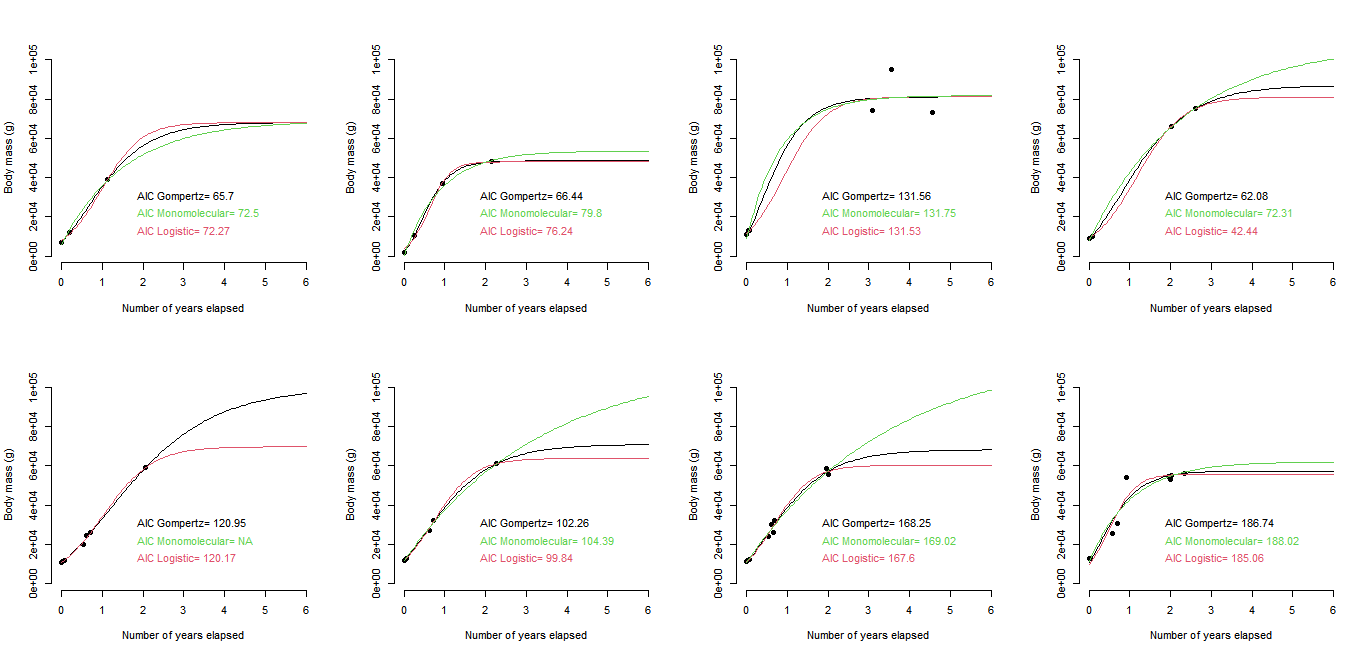


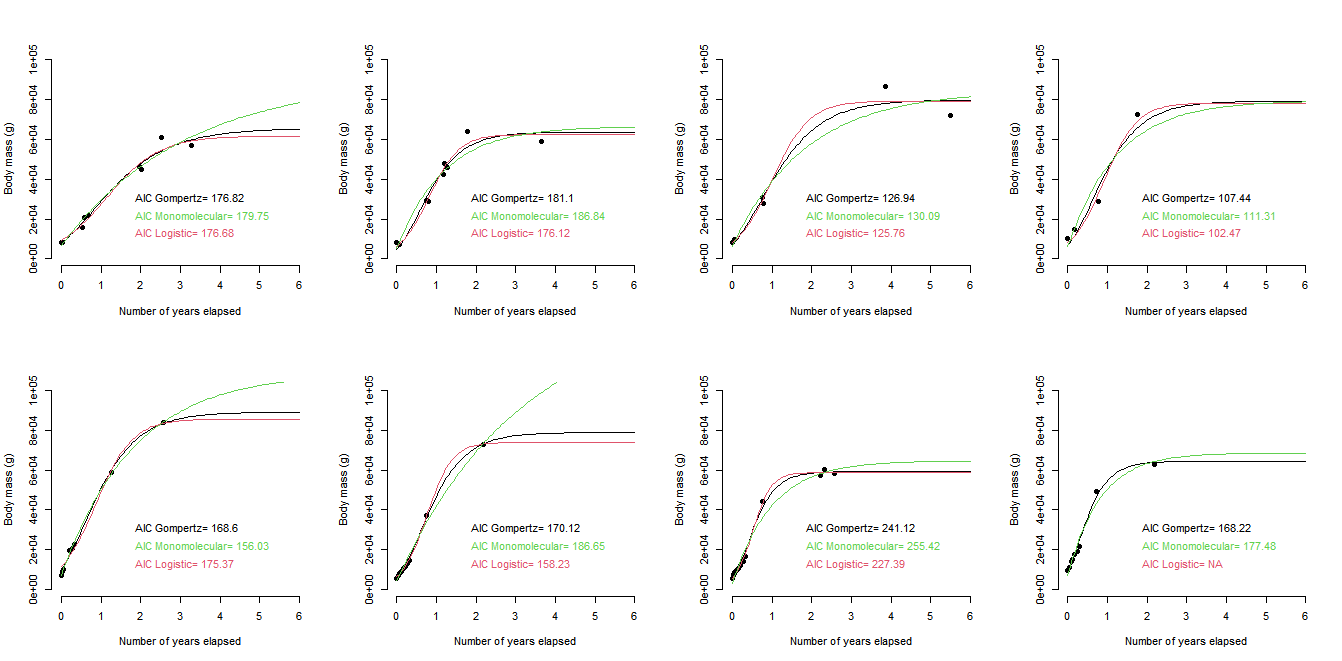


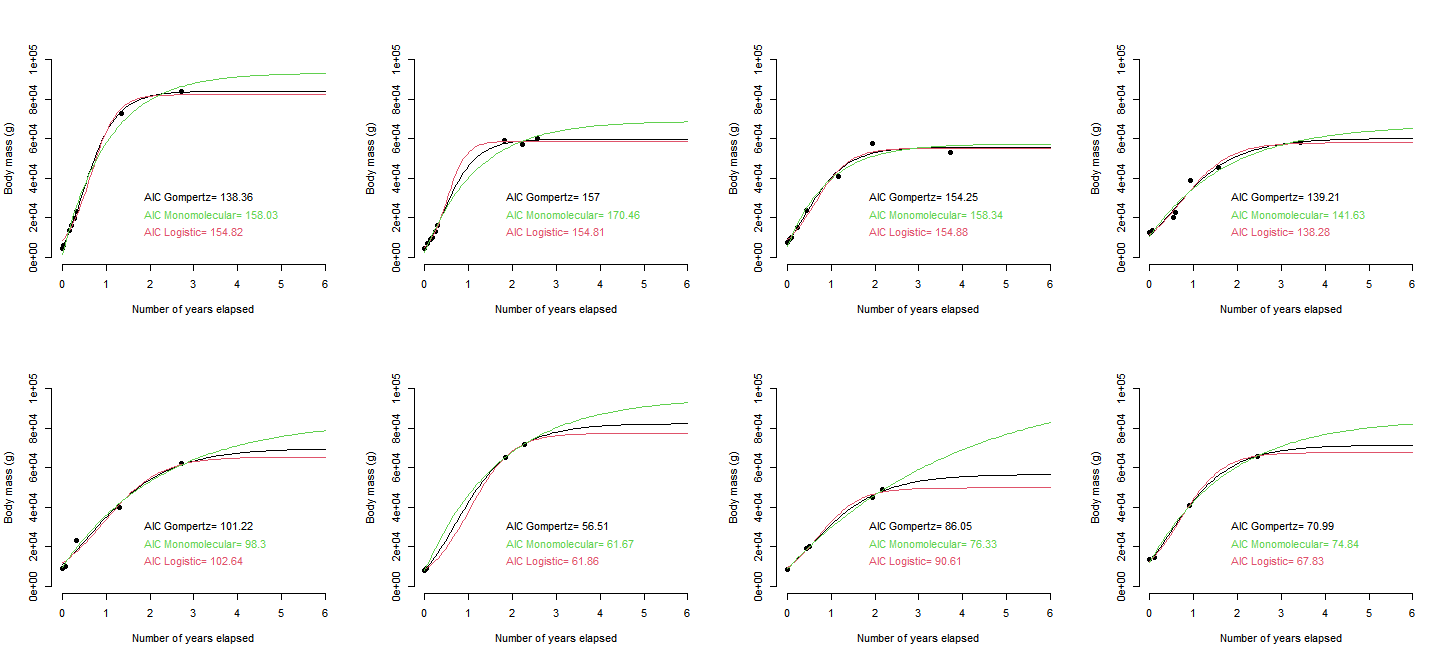


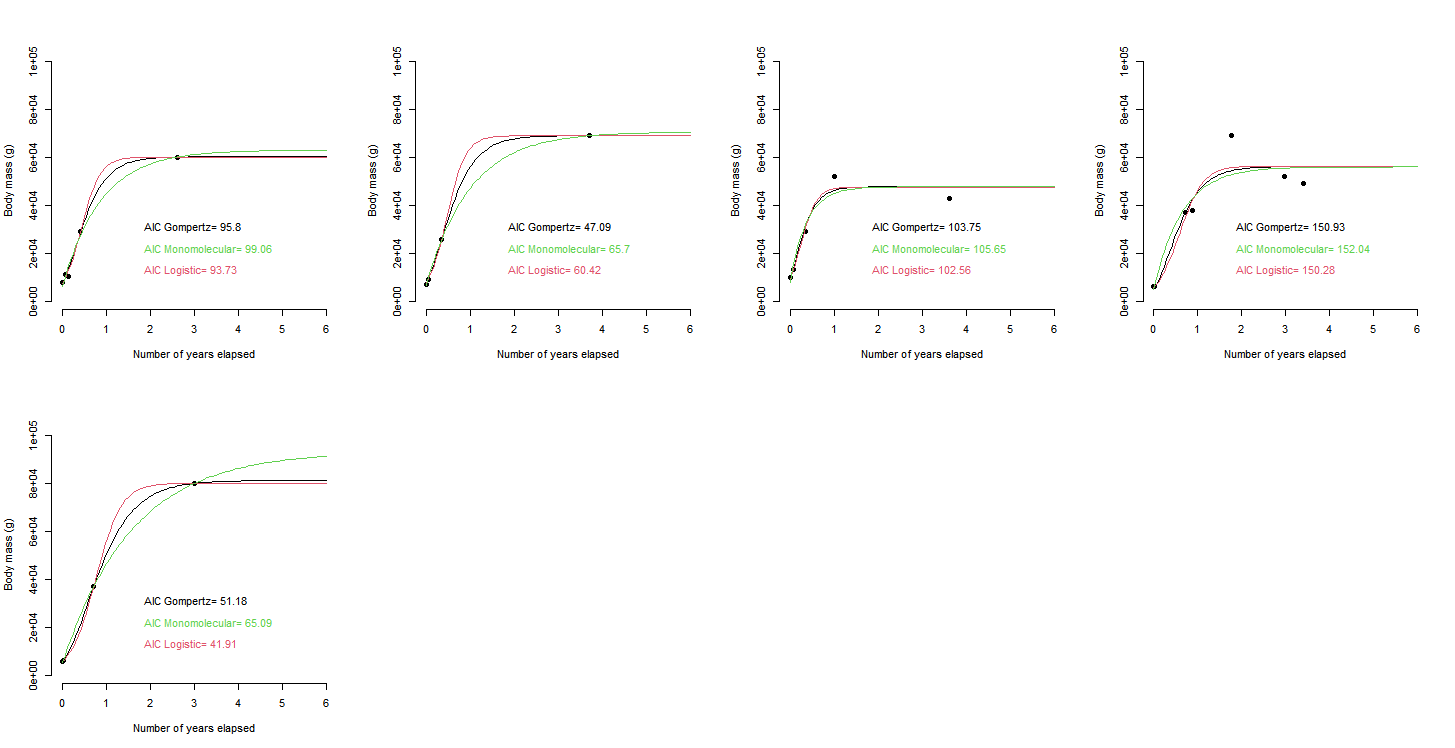


**Figure S7:** Body growth trajectories (monomolecular, Gompertz and logistic) and associated AIC for males at Chizé, from the dataset restricted to individuals with repeated measurements during both early and late in life. Each plot depicts the three models fit to a given individual’s data with points depicting per-individual observations.


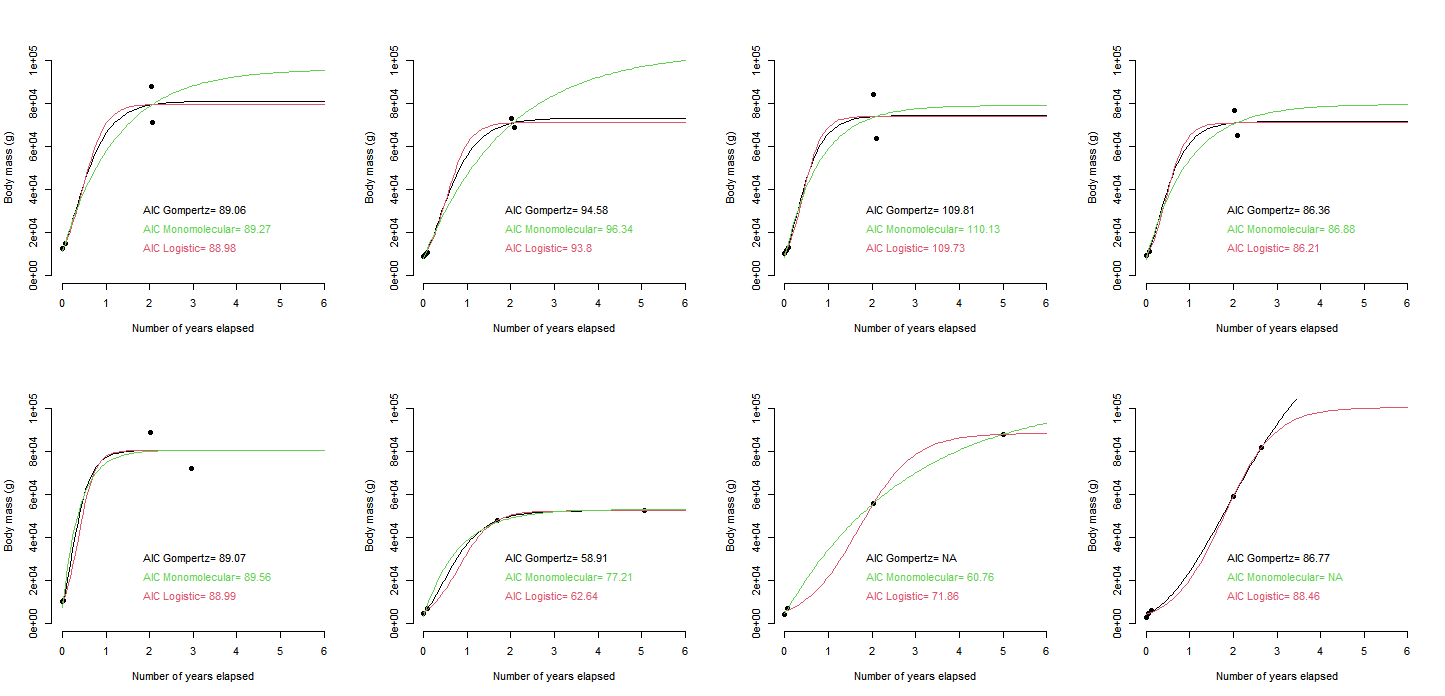


**Figure S8:** Body growth trajectories (monomolecular, Gompertz and logistic) and associated AIC for females at Chizé, from the dataset restricted to individuals with repeated measurements during both early and late in life. Each plot depicts the three models fit to a given individual’s data with points depicting per-individual observations.


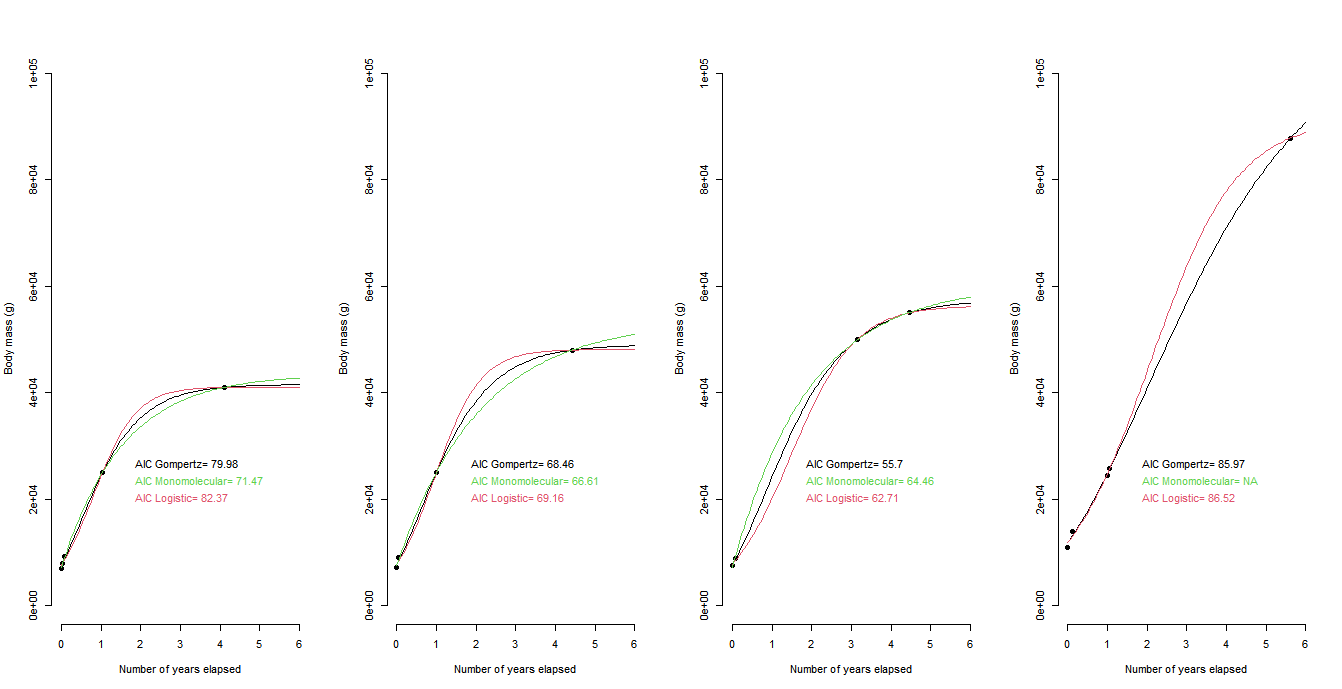


**Table S9**: Between-site and between-sex comparisons of growth parameters estimated from the best site- and sex-specific growth shape (see Table 3 for information on estimates and models). Proportional difference is reported in each case as well as t-test statistics, df and p-values. Statistically significant sex and site differences occur in bold.

| Parameter/Model | Sex differences at Chizé | Sex differences at Châteauvillain | Site differences in males | Site differences in females |
| --- | --- | --- | --- | --- |
| Asymptotic mass by Gompertz *A* | 15.12%  *t-test=0.28; df=1.59; p-value=0.81* | **37.83%**  *t-test=3.39; df=4.24; p-value=0.03* | 8.36%  *t-test=0.19; df=1.11; p-value=0.88* | 19.75%  *t-test=-0.62; df=1.07; p-value=0.64* |
| Relative growth rate by Gompertz *k* | 51.91%  *t-test=1.04; df=1.25; p-value=0.46* | **83.49%**  *t-test=-3.40; df=8.99; p-value=0.008* | 16.79%  *t-test=-0.35; df=1.13; p-value=0.78* | **68.50%**  *t-test=4.44; df=3.34; p-value=0.02* |
| Timing of maximum growth by Gompertz *t0* | 18.50%  *t-test=-0.29; df=1.94; p-value=0.80* | **46.63%**  *t-test=3.11; df=4.48; p-value=0.03* | 25.75%  *t-test=-0.42; df=1.06; p-value=0.74* | 67.71%  *t-test=-1.64; df=1.02; p-value=0.35* |
| Asymptotic mass by Logistic *A* | NA | 29.30%  *t-test=2.26; df=2.16; p-value=0.14* | 19.34%  *t-test=1.50; df=2.12; p-value=0.27* | NA |
| Relative growth rate by Logistic *k* | NA | **43.25%**  *t-test=-4.07; df=18.32; p-value=0.0007* | **62.12%**  *t-test=-8.67; df=5.47; p-value=0.0002* | NA |
| Timing of maximum growth by Logistic *t0* | NA | 36.59%  *t-test=2.57; df=2.55; p-value=0.09* | **62.35%**  *t-test=4.59; df=2.10; p-value=0.04* | NA |
| Asymptotic mass by Monomolecular *A* | NA | NA | NA | **51.16%**  *t-test=5.57; df=2.99; p-value=0.01* |
| Relative growth rate by Monomolecular *k* | NA | NA | NA | 38.30%  *t-test=-1.29; df=2.98; p-value=0.29* |
| Mean body mass at first capture by Monomolecular *I* | NA | NA | NA | 13.85%  *t-test=1.44; df=2.32; p-value=0.27* |

**Figure S10:** Kernel density estimates of asymptotic body mass *A* (in kg) for males and females in Châteauvillain (first graph) and Chizé (second graph), from the dataset restricted to individuals with repeated measurements during both early and late in life (n=37 in Châteauvillain and n=12 in Chizé). Dotted lines correspond to the sex-specific mean asymptotic body mass.


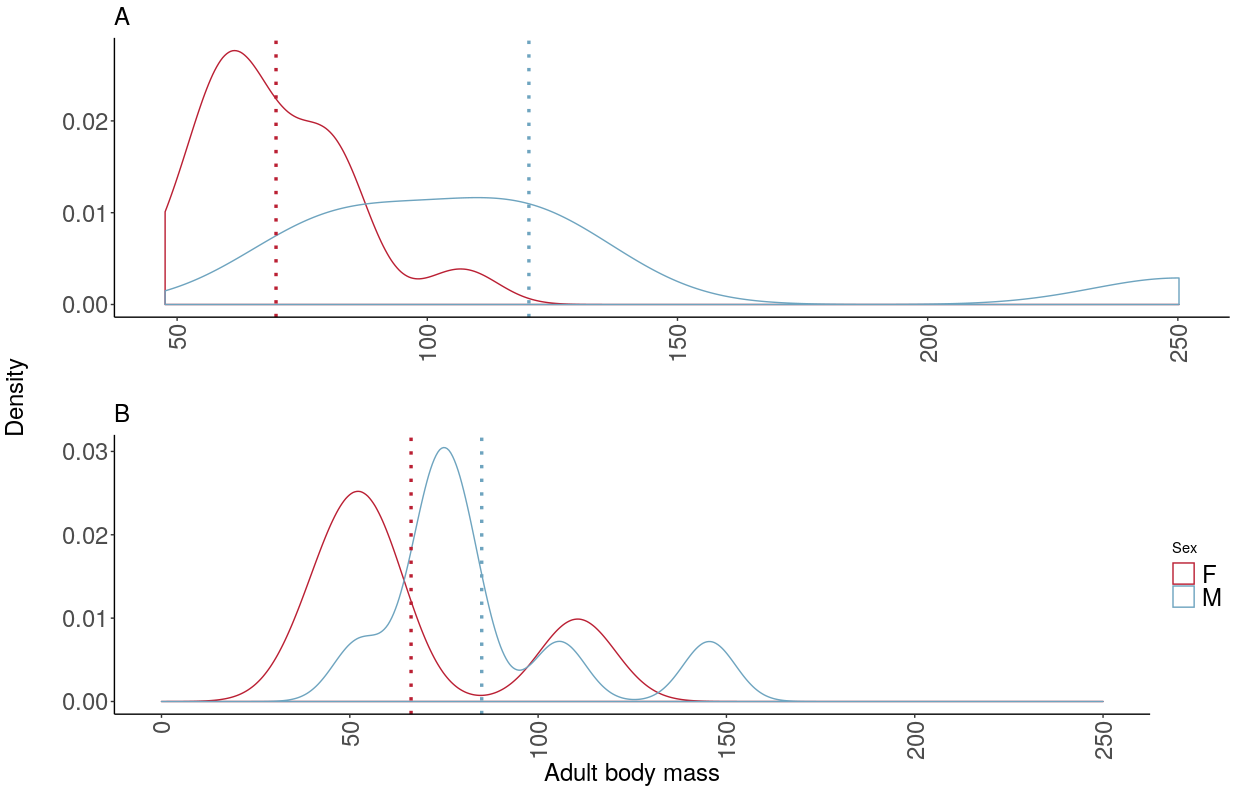


**Table S11:** Comparison of the average individual growth trajectory of wild boar fitted on the whole dataset (both sites and sexes). Models include individual random intercepts on asymptotic body mass *A*. Site and sex are included as categorical effects to test for potential sex and site effects on *A*, *t_0_* and *k*. The best model with the lowest AIC is indicated in bold.

| Model | Random effect | Categorical effect of | Categorical effect on *A* | Categorical effect on *t_0_* | Categorical effect on *k* |
| --- | --- | --- | --- | --- | --- |
|  |  |  | AIC | | |
| Gompertz | A | None | 53419.84  51545.53  51606.17  **51496.84**  51498.80 | 53419.84  51634.72  51608.67  51595.84  **51593.76** | 53419.84  51550.53  51608.67  **51506.31**  51507.99 |
| Gompertz | A | Site |  |  |  |
| Gompertz | A | Sex |  |  |  |
| Gompertz | A | Site+Sex |  |  |  |
| Gompertz | A | Site×Sex |  |  |  |
| Logistic | A | None | 51825.48  51720.22  51791.69  **51677.12**  51678.90 | 51825.48  51828.67  51792.35  51795.89  **51786.68** | 51825.48  51770.46  51790.96  **51735.11**  51737.20 |
| Logistic | A | Site |  |  |  |
| Logistic | A | Sex |  |  |  |
| Logistic | A | Site+Sex |  |  |  |
| Logistic | A | Site×Sex |  |  |  |
